# Synthesis and photooxidation of styrene copolymer bearing camphorquinone pendant groups

**DOI:** 10.3762/bjoc.8.37

**Published:** 2012-03-06

**Authors:** Branislav Husár, Norbert Moszner, Ivan Lukáč

**Affiliations:** 1 Polymer Institute, Slovak Academy of Sciences, Dúbravská cesta 9, SK-845 41 Bratislava 45, Slovakia; 2 Ivoclar Vivadent AG, Bendererstrasse 2, FL-9494 Schaan, Liechtenstein

**Keywords:** camphorquinone, 1,2-diketone, (±)-10-methacryloyloxycamphorquinone, photooxidation, polystyrene

## Abstract

(±)-10-Methacryloyloxycamphorquinone (**MCQ**) was synthesized from (±)-10-camphorsulfonic acid either by a known seven-step synthetic route or by a novel, shorter five-step synthetic route. **MCQ** was copolymerized with styrene (**S**) and the photochemical behavior of the copolymer **MCQ/S** was compared with that of a formerly studied copolymer of styrene with monomers containing the benzil (**BZ**) moiety (another 1,2-dicarbonyl). Irradiation (λ > 380 nm) of aerated films of styrene copolymers with monomers containing the **BZ** moiety leads to the insertion of two oxygen atoms between the carbonyl groups of **BZ** and to the formation of benzoyl peroxide (**BP**) as pendant groups on the polymer backbone. An equivalent irradiation of **MCQ/S** led mainly to the insertion of only one oxygen atom between the carbonyl groups of camphorquinone (**CQ**) and to the formation of camphoric anhydride (**11**) covalently bound to the polymer backbone. While the decomposition of pendant **BP** groups formed in irradiated films of styrene copolymers with pendant **BZ** groups leads to crosslinking, only small molecular-weight changes in irradiated **MCQ/S** were observed.

## Introduction

**Scheme 1:**
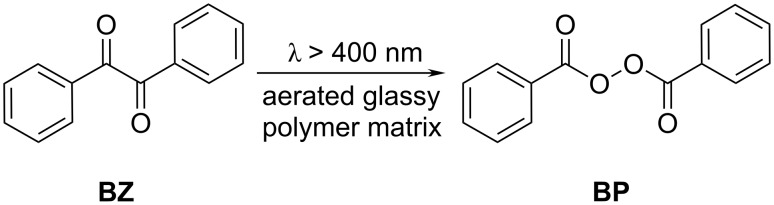
Photoperoxidation of **BZ** in an aerated glassy polymer matrix.

Camphorquinone (**CQ**) in the presence of H-atom donors such as ethers (H abstraction), or more efficiently tertiary amines (electron/proton transfer), is known to be an effective photoinitiator for curing methacrylate-based dental restorative resins [1–9]. **CQ** photochemistry in solution in the absence of oxygen [10–16] and in the presence of oxygen [10,13,17–20] has been studied extensively. In an inert atmosphere, the excited *n*→π* triplet state of the carbonyl group of **CQ** abstracts an H-atom from a hydrogen donor. The two primarily formed radicals undergo subsequent reactions leading to photoproducts [21]. The rate-determining step in photoinitiation by **CQ**/amine is hydrogen transfer by the excited *n*→π* triplet state of the carbonyl group of **CQ** from the alkylamino group [8–9]. The photochemistry of the low molecular **CQ** in the polystyrene (**PS**) film was the subject of previous studies [21–22]. It is reasonable to compare the photochemical properties of polymer-bound **CQ** with a polymer matrix containing another well-studied 1,2-dicarbonyl compound, namely benzil (**BZ**). **BZ** can be converted almost quantitatively to benzoyl peroxide (**BP**) in an aerated polymer matrix by irradiation at λ > 400 nm (Scheme 1) [23–24]

The pendant **BZ** groups of the styrene copolymers may be transformed into pendant **BP** groups [25–26]. Subsequent decomposition of **BP** pendant groups results in the formation of highly crosslinked films [27–30].

Owing to the interesting properties of styrene copolymers formed from **BZ**-containing monomers, a **CQ**-bearing monomer (±)-10-methacryloyloxycamphorquinone (**MCQ**) (another monomer with a 1,2-dicarbonyl moiety) was prepared and copolymerized with styrene to give **MCQ/S** copolymer bearing **CQ** pendant groups. Enantiopure **MCQ** is known from the literature [31]. The goal of this work was to prepare a more easily accessible racemic **MCQ** and to compare the photochemistry of **MCQ/S** with that of low molecular **CQ** in **PS** films in the presence of oxygen.

## Results and Discussion

### Synthesis of MCQ

**Scheme 2:**
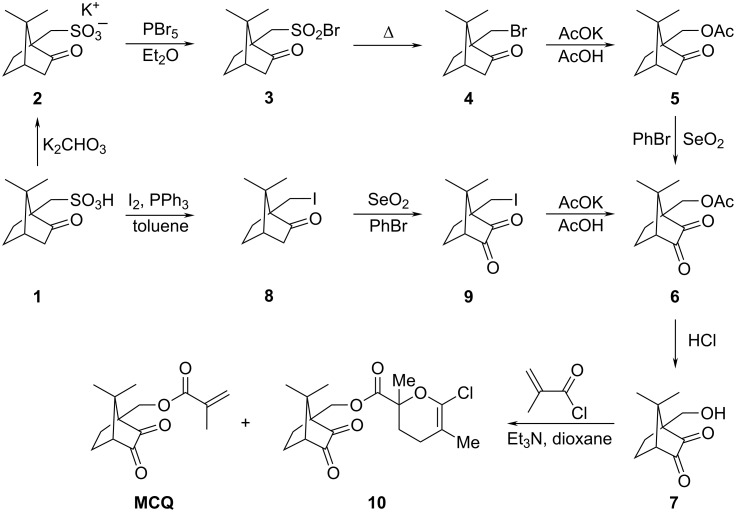
Synthesis of **MCQ** from (±)-10-camphorsulfonic acid (**1**). Only one enantiomer of each compound is depicted.

Racemic **MCQ** was synthesized in seven steps from (±)-10-camphorsulfonic acid (**1**) as a starting material in 18% overall yield (Scheme 2, see Supporting Information File 1 and Supporting Information File 2 for full experimental data). Total synthesis of (1*R*)-10-methacryloyloxycamphorquinone from (1*S*)-10-camphorsulfonic acid [31] as well as of enantiopure stable intermediates **2**–**7** [8,32–34] are known. Though the physiological activity of optically active compounds depends on their configuration, the photochemical activity of **CQ** does not depend on the configuration. Without the need for an enantiopure product, the reaction economics could be improved both by the use of a cheaper racemic starting material and by a simplified multistep synthesis.

A shorter alternative synthetic route to **6** from **1** was proposed (Scheme 2, see Supporting Information File 1 and Supporting Information File 2 for full experimental data). (±)-10-Iodocamphor (**8**) was prepared in one step from **1** with iodine and PPh_3_ in toluene under reflux in 39% yield (lit. [35] 85% yield). Compound **8** was selectively oxidized with SeO_2_ in bromobenzene under reflux to (±)-10-iodocamphorquinone (**9**) in 84% yield. A suspension of **9** in anhydrous acetic acid with freshly molten potassium acetate was heated to 170 °C for 8 h to provide **6** in 37% yield.

The advantage of this synthetic pathway lies in the reduction of synthetic steps as well as avoiding the use of rather expensive PBr_5_. Although we obtained **MCQ** in overall yield of only 5%, optimization of conditions will likely provide a higher value. For example, iodocamphor **8** was previously obtained in 85% yield (much better than the 39% yield expressed here). Low yield in this step is blamed on the poor quality of reactants. The yield of **6** from **9** was lower due to the formation of side products. Thus, iodocamphor **8** should be transformed first to **5** and afterwards to **6**.

All attempts to transform **9** directly to **MCQ** were unsuccessful. For example, such a transformation was tested by stirring compound **9** at ambient temperature with the following reagents: Methacrylic acid/Cs_2_CO_3_/DMF, methacrylic acid/DBN/benzene, methacrylic acid/NaH/*n*-hexane, methacrylic acid/NaHCO_3_/DMF, potassium methacrylate/acetone, and silver methacrylate/toluene.

From the last step of the **MCQ** synthesis, a side-product **10** was isolated and identified. It was previously reported that a commercial sample of methacryloyl chloride contains its oxa-Diels–Alder dimer, responsible for the formation of **10** [36]. This side-product could be avoided by using freshly distilled methacryloyl chloride. A significant difference in the determined melting points of the racemates and enantiomers of **6**, **7**, and **MCQ** is caused by the configuration of these compounds (Table 1). The decrease of melting point of the racemate is 10–12 °C in the case of **6** and 43–45 °C in the case of **MCQ** compared to the pure enantiomer. Racemic **7** melts over a broad range of temperatures in contrast to enantiomeric **7**, which has a sharp melting point. The compounds in this work are racemates, but the corresponding compounds in the literature are pure enantiomers. The racemates can crystallize as a racemic mixture (lower mp), as a racemic compound (lower or higher mp), or rarely as a racemic solid solution (slightly lower or higher mp).

**Table 1:** Values of melting points of synthesized racemic camphor derivatives and corresponding pure enantiomers (*R* or *S*) from the literature.

Compound	Melting point of racemate (°C)	Melting point of enantiomer (°C)	Configuration of enantiomer (*R* or *S*)	Reference

**4**	77–78	78	*S*	[35]
**4**	77–78	78	*R*	[32]
**4**	77–78	76–78	*R*	[8]
**6**	76–78	88	*R*	[33]
**6**	76–78	87–90	*R*	[8]
**7**	≈100–255	201–203	*R*	[8]
**7**	≈100–255	205	*R*	[34]
**8**	67–72	71	*S*	[35]
**MCQ**	46–49	91–92	*R*	[31]

### Polymerization

As introduced in Supporting Information File 1, the copolymer **MCQ/S** was synthesized by copolymerization of styrene (99.54 mol %) and **MCQ** (0.46 mol %), initiated by AIBN at 60 °C and resulting in 10% conversion. FTIR spectroscopy was used to estimate the content of **CQ** units in the **MCQ/S** copolymer by interpolation of the peak area of the carbonyl band (1740–1790 cm^−1^) using a calibration curve consisting of five different concentrations of **MCQ** in CCl_4_ solution. UV–vis spectroscopy was used in a similar way by interpolation of the *n*→π* peak area (390–510 nm) using a calibration curve. The content of **MCQ** units in **MCQ/S** copolymer was determined to be 0.72 mol % from FTIR and 0.62 mol % from UV–vis. **MCQ** is therefore more reactive than styrene, which is in agreement with the copolymerization parameters of structurally similar monomers.

#### Photooxidation of MCQ/S

**Scheme 3:**
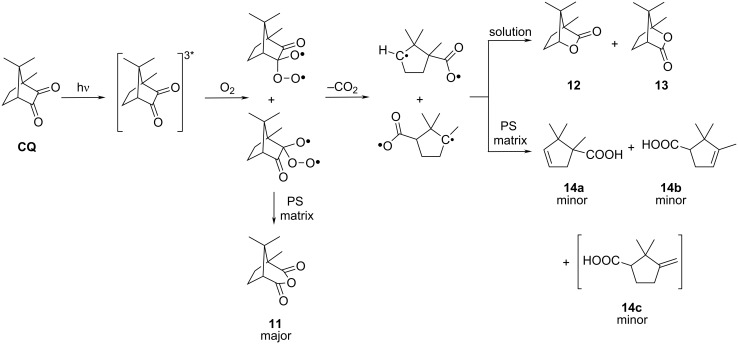
Photooxidation of **CQ** in aerated glassy **PS** matrix.

Since it is difficult to follow the structural changes of the **CQ** structures of the **MCQ/S** copolymer during photochemical transformation, an analogous study with low molecular **CQ** doped in a **PS** matrix was first performed. Elucidation of the structures of low molecular photoproducts was conveniently followed by spectral methods [22] and by isolation from the polymer matrix followed by spectral identification [21]. The results of the **CQ** photooxidation in **PS** are summarized in Scheme 3 [21].

The addition of molecular oxygen to the excited *n*→π* triplet state of ketones and 1,2-diketones to form 1,4-biradicals is a generally accepted mechanism, which has been theoretically treated and reviewed [37]. The oxygen atom released during the formation of **11** can oxidize another molecule of **CQ** to **11**. It is likely that common biradical intermediates are responsible for the formation of lactones **12** and **13** in solution [10] and acids **14a** and **14b** formed in the **PS** matrix [21]. The intramolecular recombination of biradical intermediates is favored in benzene solution. However, in glassy **PS** matrix the intramolecular abstraction of a hydrogen atom and formation of a double C=C bond occurs. The glassy polymer matrix should retard the intramolecular recombination of biradicals.

**Figure 1:**
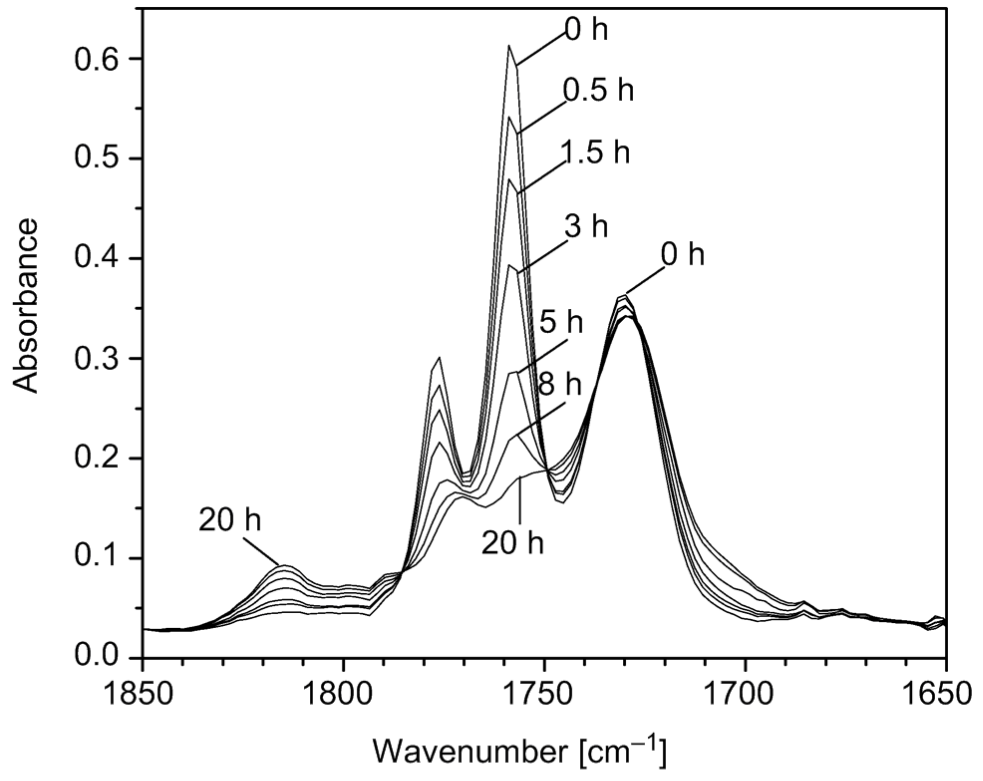
FTIR spectra of **MCQ/S** film after irradiation in a carousel for the indicated periods. Spectrum of **PS** film was subtracted.

**Figure 2:**
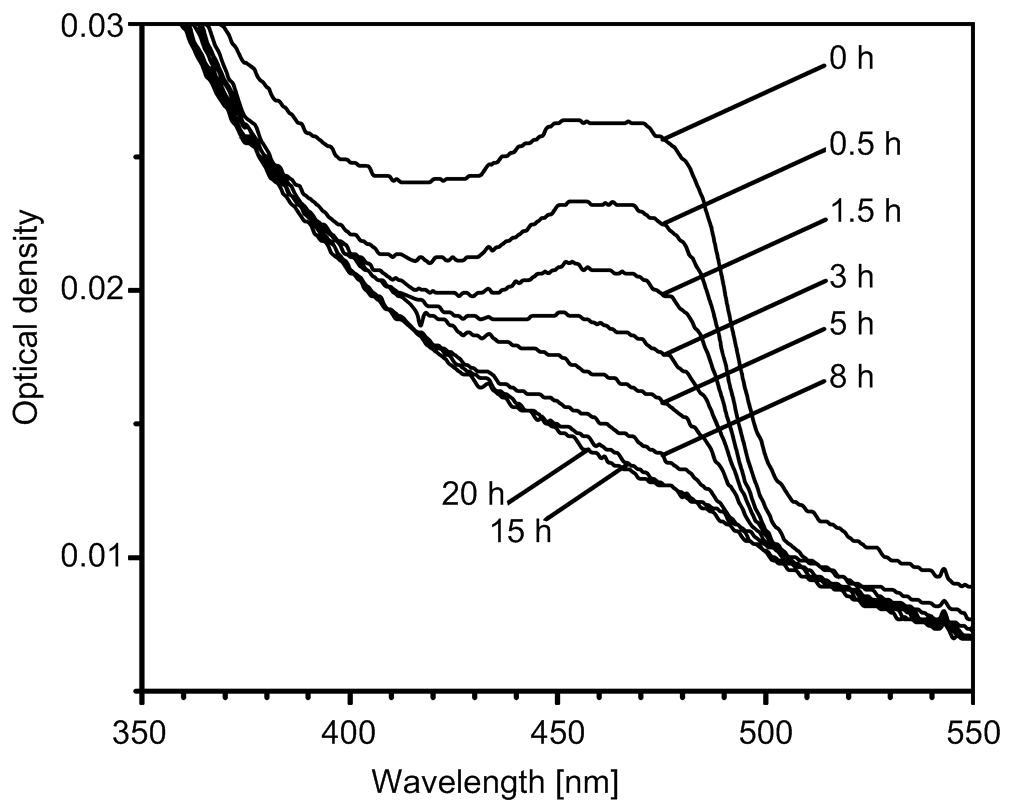
UV–vis spectra of **MCQ/S** film after irradiation in a carousel apparatus for the indicated periods.

During the irradiation of **MCQ/S** film at λ > 380 nm in air, the changes were followed by FTIR (Figure 1) and UV–vis (Figure 2) spectroscopy. The evolution of both spectra of low molecular **CQ** doped in the **PS** matrix and that of copolymer **MCQ/S** during irradiation (beside ester carbonyl absorption in FTIR spectra) are similar. Absorption bands of the **CQ** 1,2-dicarbonyl group vibrations (1776, 1759 cm^−1^) decreased quantitatively. This decrease is accompanied by the formation of bands at 1815 and 1770 cm^−1^ assigned to anhydride **11** (Figure 1). Increased absorption near 1700 cm^−1^ was assigned to acids **14**. After thermal treatment of the irradiated **MCQ/S** film at 90 °C for 2 h, no change was observed by FTIR spectroscopy. This signifies that no thermally unstable eight-member ring peroxide (in analogy with **BP** formed from **BZ** as shown in Scheme 1) was present in the **PS** matrix after irradiation. FTIR vibration bands for such cyclic diacylperoxide would be expected to be found near 1800 cm^−1^. Also in UV–vis spectra of the **MCQ/S** film after irradiation (Figure 2), complete reduction of the *n*→π* absorption band of the 1,2-dicarbonyl group of the **CQ** structure is seen.

The GC–MS results [21] allow the rough estimation of the distribution of **CQ** photooxidation products in **PS** from the GC record. About 54% of the whole area corresponds to **11** and its secondary products formed during isolation (secondary products are not formed in the **MCQ/S** film during irradiation). 16% corresponds to **14a**–**c** and the remaining 30% are unidentified compounds. A similar distribution of products is most probably also present in the photooxidized **MCQ/S** copolymer.

Whereas the previously published irradiation of styrene copolymers with the monomers containing **BZ** (*M*_n_ = 84000, PD = 1.8) with 0.54 mol % of the **BZ**-containing component led to extensive crosslinking with 69% gel fraction [21], **MCQ/S** with 0.72 mol % of **CQ** moieties did not reach the gel point under the same conditions of irradiation; **MCQ/S** film retained its solubility in organic solvents. **PS** equivalent molar masses were then determined by GPC before and after irradiation. The molecular weights and polydispersities of the irradiated copolymer (*M*_n_ = 190000, PD = 2.7) were higher than those of the initial copolymer (*M*_n_ = 170000, PD = 2.0). Therefore, some recombination of macroradicals occurred during irradiation. However, the crosslinking of styrene copolymers with the monomers containing **BZ** is significantly more efficient than those of **MCQ/S**.

**Scheme 4:**
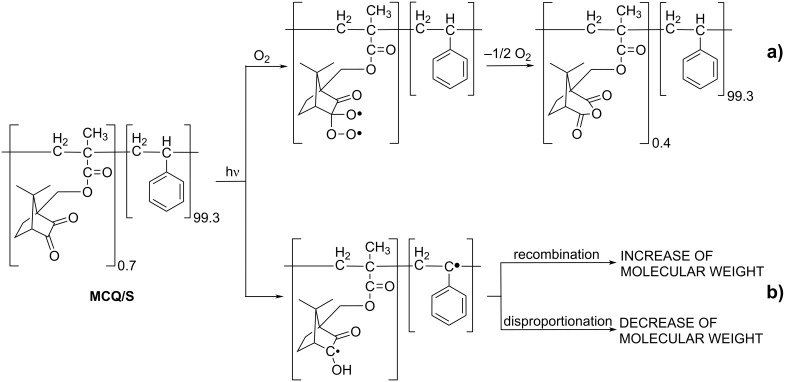
Proposed mechanism of **MCQ/S** photochemistry.

While in the case of styrene copolymers with the monomers containing **BZ** decomposition of pendant **BP** groups produces only one polymeric benzoyloxy radical that is efficient in crosslinking, in the **MCQ/S** copolymer two acyloxy radicals could be produced from the postulated covalently bound cyclic diacylperoxide and could cause extensive crosslinking. As the changes in the chemical and molecular weights show, the mechanism of **MCQ/S** crosslinking (increase of molecular weight) is clearly different from that in the presence of **BZ** structures. Hence, the covalently bound cyclic diacylperoxide as an intermediate is most probably not formed. Similar to **BZ**, the *n*→π* triplet state of the **CQ** structure may also add molecular oxygen to form a 1,4-biradical. In the case of the **BZ** structures, formation of the 1,4-biradical is followed by **BP** formation. In comparison, **CQ** structures react with oxygen forming 1,4-biradicals, which decompose to camphoric anhydride **11** structures covalently bound to the polymer backbone (Scheme 4a). Liberated atomic oxygen during the formation of covalently bound **11** may oxidize another pendant **CQ** unit to the covalently bound **11**. Cyclopentenecarboxylic acid **14** structures shown in Scheme 3 according to the low molecular study [21] were formed to a much lesser extent. Since the phototransformation of **CQ** to **11** is not quantitative, *n* + *m* = 99.7; the remaining 0.3 consists of cyclopentenoic acids **14** and other unknown photoproducts.

The increase of the molecular weight of **MCQ/S** during irradiation under low oxygen conditions is induced primarily by an abstraction of hydrogen from the polymer backbone by the **CQ** structure in the triplet state under the formation of ketyl and alkyl radicals. The formed macroradicals can recombine and/or disproportionate (Scheme 4b). Recombination of macroradicals leads to an increase of the molecular weight and polydispersity. As previously shown, oxygen increases the consumption rate of **CQ** added to **PS** and lowers the rate of decrease of the molecular weight of **PS** [22]. Therefore in simultaneous photooxidation and photoreduction (Scheme 4) in the presence of oxygen, the intramolecular photooxidation of **CQ** has almost no effect on the molecular weight [21]. In the case of **MCQ/S**, photoreduction forms macroradicals that recombine causing an increase in the molecular weight.

## Conclusion

Upon irradiation of **MCQ/S** copolymer film by light with λ > 380 nm in air, the **CQ** structure in the copolymer was transformed mainly to pendant camphoric anhydride **11** structures. Also cyclopentenecarboxylic acid **14** structures covalently bound to copolymer backbone were identified to a minor extent. No cyclic camphordiacyl peroxide as an intermediate of the **CQ** photooxidation was found. Crosslinking of **MCQ/S** is inefficient compared to the case of styrene copolymers with monomers containing the **BZ** moiety.

Monomer **MCQ** (racemate) was synthesized from camphorsulfonic acid in seven steps by a known synthetic pathway for optically active **MCQ**. As an improvement, an alternative five-step synthesis of **MCQ** was proposed as well.

## Supporting Information

Supporting information contains detailed experimental data for the synthesis of the compounds **2**–**10**, **MCQ**, and copolymer **MCQ/S**, irradiation conditions, and NMR spectra of **5**–**10** and **MCQ**.

File 1 Experimental part.

File 2 NMR spectra of compounds **5**–**10** and **MCQ**.
