# Supplementary material for: Synthesis and photooxidation of styrene copolymer bearing camphorquinone pendant groups
Source: Beilstein J Org Chem. 2012 Mar 6;8:337–43. doi: 10.3762/bjoc.8.37 (PMC3326610; doi:10.3762/bjoc.8.37)
Supplement: File 1 — Experimental part. [file Beilstein_J_Org_Chem-08-337-s001.pdf]

Supporting Information File 1

for

Synthesis and photooxidation of styrene copolymer bearing camphorquinone pendant groups

Branislav Husár*[1], Norbert Moszner[2] and Ivan Lukáč[1]

Address: [1]Polymer Institute, Slovak Academy of Sciences, Dúbravská cesta 9, SK-845 41 Bratislava 45, Slovakia and [2]Ivoclar Vivadent AG, Bendererstrasse 2, FL-9494 Schaan, Liechtenstein

Email: Branislav Husár* - branislav.husar@savba.sk; Norbert Moszner - norbert.moszner@ivoclarvivadent.com; Ivan Lukáč - ivan.lukac@savba.sk

* Corresponding author

Experimental part

Table of contents

Materials S2

Characterization S2

Syntheses S3

Polymerization S10

Irradiation S11

Experimental

Materials

(±)-10-Camphorsulfonic acid, phosphorus pentabromide, methacryloyl chloride, anhydrous dioxane (Aldrich), triethylamine (Fluka), iodine, triphenylphosphine (Lachema), and all solvents (p.a.) were used as received. Selenium dioxide (Reachim) was resublimed from a nitric acid suspension. Potassium acetate was freshly molten before use. Acetic acid was dried over P_2O_5 and distilled. Diethyl ether was dried over anhydrous Na_2SO_4 for several days, filtered and dried with a sodium wire and distilled. Xylene was dried with a sodium wire and distilled. Stabilized styrene (Aldrich) was washed with diluted sodium hydroxide solution and water, dried with anhydrous $MgSO_4$ and distilled. Azobisisobutyronitrile (AIBN) (Fluka) was recrystallized from methanol.

Characterization

^{1}H NMR (600 MHz) and ^{13}C NMR (150 MHz) spectra were recorded with a Varian Inova 600 spectrometer in $CDCl_3$, with the solvent signal as a reference. Chemical shifts are given in the δ-scale (ppm), with coupling constants *J* given in Hz. COSY, HSQC, and HBMC sequences were used for an assignment of signals. FTIR spectra were recorded on a Nicolet Impact 8700 FT spectrophotometer. UV–vis absorption spectra were measured on a Shimadzu 1650PC spectrophotometer (Japan). Mass spectra were recorded with a Hewlett Packard 5989B instrument. Melting points were determined by a hot-stage apparatus (Nageman, Germany) and are uncorrected. **PS** equivalent molar masses were estimated by GPC with THF as a mobile phase, series-connected PSS SDV 500 Å and PSS SDV 10^5 Å columns (d = 8 mm, l =

300 mm), Knauer 64 pump (1 mL min^{-1}, 3.3–3.4 MPa), and RI WATERS 410 detector. Loop 100 μL and polymer concentration 2 mg mL^{-1} were used.

Syntheses

Syntheses of the compounds **6**, **7**, **9**, and **MCQ** were carried out in darkness or under illumination at a wavelength at which **CQ** does not absorb.

Potassium (±)-10-camphorsulfonate (2)

A solution of (±)-10-camphorsulfonic acid (**1**) (250 g, 1.08 mol) in water (300 mL) was neutralized with K_2CO_3 (79.3 g, 0.57 mol). Water was evaporated under reduced pressure and acetone was added and the salt was filtered. The residue was evaporated and combined with filtered salt. Pulverized **2** was dried for several days in a vacuum desiccator over P_2O_5 until a constant weight was measured and was used without further purification.

(±)-10-Bromosulfonylcamphor (3)

A stirred suspension of **2** (62.0 g, 0.229 mol) in anhydrous diethyl ether (300 mL) under argon was cooled to −60 °C. PBr_5 (100 g, 0.232 mol) was added at once. The cooling bath was removed and the reaction mixture was stirred for 1 h at ambient temperature and then heated under reflux for 3 h. The reaction mixture was poured into a mixture of ice (600 g) and water (600 mL). The ethereal layer and the white solid were separated and the aqueous layer was extracted with diethyl ether (2 × 100 mL). The combined ethereal layers were washed with water (50 mL), dried over Na_2SO_4 and evaporated to give a yellow solid **3** (58.0 g, 86%), which was used without further purification.

^1H NMR ($CDCl_3$): δ = 4.47 (d, *J* = 14.4 Hz, 1H, 10-H_a), 3.92 (d, *J* = 14.4 Hz, 1H, 10-H_b), 2.54–2.39 (m, 2H, CH_2), 2.18–2.04 (m, 2H, CH_2), 1.99 (d, *J* = 18.3 Hz, 1H, CH_2), 1.84–1.74 (m, 1H, CH_2), 1.49 (ddd, *J* = 12.3, 9.3, 3.6 Hz, 1H, CH_2), 1.14 (s, 3H, 8-H), 0.93 (s, 3H, 9-H)

(±)-10-Bromocamphor (4)

A solution of **3** (58.0 g, 0.196 mol) in anhydrous xylene was kept in darkness over $CaCl_2$ (10 g) overnight. The mixture was heated under reflux for 3 h under argon. The solid part was removed by filtration and the solution was concentrated. After a distillation (75–80 °C and 0.13 Torr) followed by a crystallization from methanol, a white solid **4** (38.3 g) was obtained in 84% yield, mp 77–78 °C.

^1H NMR ($CDCl_3$): δ = 3.62 (d, *J* = 10.8 Hz, 1H, 10-H_a), 3.41 (d, *J* = 10.8 Hz, 1H, 10-H_b), 2.41 (ddd, *J* = 18.2, 4.9, 2.9 Hz, 1H, 3-H_{eq}), 2.17–2.09 (m, 2H, 4-H + 6-H), 2.08–1.97 (m, 1H, 5-H_{eq}), 1.91 (d, *J* = 18.2 Hz, 1H, 3-H_{ax}), 1.64–1.51 (m, 1H, 6-H), 1.41 (ddd, *J* = 12.3, 9.3, 3.4 Hz, 1H, 5-H_{ax}), 1.11 (s, 3H, 8-H), 0.95 (s, 3H, 9-H)
^{13}C NMR ($CDCl_3$): δ = 215.3 (C-2), 60.2 (C-1), 48.1 (C-7), 43.9 (C-4), 42.9 (C-3), 29.3 (C-10), 27.6 (C-6), 26.7 (C-5), 20.4 (C-8), 20.2 (C-9);
FTIR (ATR): $\tilde{\nu}$ /cm^{-1} = 1746 (C=O).

(±)-10-Acetoxycamphor (5)

A suspension of **4** (30.0 g, 130 mmol) in anhydrous acetic acid (60 g, 57 mL) and freshly molten potassium acetate (90.0 g, 917 mmol) was heated at 150 °C for 17 h. The cooled reaction mixture was dissolved in water (200 mL), neutralized with an aqueous solution of Na_2CO_3 and extracted with diethyl ether (3 × 100 mL). Combined

ethereal layers were washed with water (50 mL), dried over Na_2SO_4 and concentrated under reduced pressure. Distillation (75–80 °C and 0.13 Torr) afforded **5** (26.1 g, 96%) as a colorless oil.

^{1}H NMR ($CDCl_3$): δ = 4.28 (d, *J* = 12.3 Hz, 1H, 10-H_a), 4.23 (d, *J* = 12.3 Hz, 1H, 10-H_b), 2.43 (ddd, *J* = 18.2, 4.7, 2.3 Hz, 1H, 3-H_{eq}), 2.10–1.93 (m, 7H, 4-H + 5-H_{eq} + 6-H + C$\underline{H}_3$─C═O + 3-H_{ax}), 1.48–1.34 (m, 2H, 5-H_{ax} + 6-H), 1.05 (s, 3H, 8-H), 0.98 (s, 3H, 9-H);

^{13}C NMR ($CDCl_3$): δ = 216.2 (C-2), 171.0 (CH_3─$\underline{C}$═O), 60.5 (C-10), 60.1 (C-1), 47.0 (C-7), 43.9 (C-4), 43.3 (C-3), 26.6 (C-6), 25.5 (C-5), 20.9 (CH_3), 20.7 (CH_3), 19.8 (CH_3);

FTIR (neat): $\tilde{\nu}$ /cm^{-1} = 1747 (C═O), 1744 (C═O).

(±)-10-Acetoxycamphorquinone (6) from 5

A suspension of **5** (44.0 g, 209 mmol), freshly resublimed SeO_2 (29.5 g, 266 mmol) in bromobenzene (250 mL) was refluxed for 17 h. A cooled reaction mixture was concentrated and filtered through a short silica column to remove arose selenium, then the column was washed with ethyl acetate and the solution was concentrated. The residue was purified by column chromatography (silica gel, ethyl acetate/petroleum ether 1:3) to afford **6** as a yellow solid in 67% yield (31.3 g), mp 76–78 °C.

^{1}H NMR ($CDCl_3$): δ = 4.40 (d, *J* = 12.6 Hz, 1H, 10-H_a), 4.36 (d, *J* = 12.6 Hz, 1H, 10-H_b), 2.64 (d, *J* = 5.0 Hz, 1H, 4-H), 2.30–2.10 (m, 2H, CH_2), 2.08 (s, 3H, C$\underline{H}_3$─C═O), 1.76–1.63 (m, 2H, CH_2), 1.17 (s, 3H, 8-H), 1.08 (s, 3H, 9-H);

^{13}C NMR ($CDCl_3$): δ = 201.6 (C=O), 201.3 (C=O), 170.6 (CH_3–$\underline{C}$=O), 60.8 (C-1), 59.3 (C-10), 58.5 (C-4), 42.8 (C-7), 25.8 (CH_2), 22.0 (CH_3), 21.8 (CH_2), 20.8 (CH_3), 18.2 (CH_3);

FTIR (ATR): $\tilde{\nu}$/cm^{-1} = 1771 (C=O), 1747 (C=O), 1736 (C=O);

UV–vis ($CHCl_3$): λ_{max}/nm (ε/mol^{-1} dm^3 cm) = 468 (40).

(±)-10-Hydroxycamphorquinone (7)

A solution of **6** (3.1 g, 14 mmol) in 20% HCl was heated under reflux for 2 h. The cooled reaction mixture was neutralized with an aqueous solution of Na_2CO_3 and extracted with ethyl acetate (3 × 50 mL). The organic layer was dried over $CaCl_2$ and concentrated. The residue was purified by column chromatography (silica gel, ethyl acetate/petroleum ether 1:2) to afford **7** as a yellow solid in 91% yield (2.3 g), which is a sublimating compound with an extremely wide melting range ≈100–255 °C determined in a sealed capillary.

^1H NMR ($CDCl_3$): δ = 4.01 (d, J = 12.2 Hz, 1H, 10-H_a), 3.87 (d, J = 12.2 Hz, 1H, 10-H_b), 2.63 (d, J = 4.9 Hz, 1H, 4-H), 2.29–2.01 (m, 2H, 5-H + 6-H), 1.82–1.64 (m, 2H, 5-H + 6-H), 1.15 (s, 3H, 8-H), 1.10 (s, 3H, 9-H);

^{13}C NMR ($CDCl_3$): δ = 205.0 (C-2), 202.2 (C-3), 62.9 (C-1), 59.2 (C-10), 58.7 (C-4), 42.5 (C-7), 26.0 (C-6), 22.3 (C-9), 21.9 (C-5), 17.9 (C-8);

FTIR (ATR): $\tilde{\nu}$/cm^{-1} = 3330 (O–H), 1769 (C=O), 1747 (C=O);

UV–vis ($CHCl_3$): λ_{max}/nm (ε/mol^{-1} dm^3 cm) = 470 (41).

(±)-10-Iodocamphor (8)

To a solution of **1** (46.5 g, 0.2 mol) and triphenylphosphine (262.5 g, 1 mol) in toluene (1 L) was added iodine (152.5 g, 0.6 mol) and the mixture was heated under reflux for 30 h. The reaction mixture was cooled to ambient temperature and extracted with water (2 × 400 mL) and 3% aqueous solution of $Na_2S_2O_3$ (400 mL), and the organic layer was dried over Na_2SO_4 overnight and concentrated. Distillation (120–150 °C and 0.13 Torr) followed by crystallization from methanol yielded 21.6 g (39%) of colorless solid **8**, mp 67–72 °C.

^{1}H NMR ($CDCl_3$): δ = 3.31 (d, *J* = 10.7 Hz, 1H, 10-H_a), 3.12 (d, *J* =10.4 Hz, 1H, 10-H_b), 2.40 (ddd, *J* = 18.3, 4.9, 1.8 Hz, 1H, 3-H_{eq}), 2.19–2.14 (m, 1H, 4-H), 2.06–1.95 (m, 2H, 5-H_{eq} + 6-H), 1.91 (d, *J* = 18.3 Hz, 1H, 3-H_{ax}), 1.61 (t, *J* = 9.2 Hz, 1H, 6-H), 1.40 (t, *J* = 9.2 Hz, 1H, 5-H_{ax}), 1.08 (s, 3H, 8-H), 0.90 (s, 3H, 9-H);
^{13}C NMR ($CDCl_3$): δ = 215.2 (C-2), 59.0 (C-1), 48.3 (C-7), 44.0 (C-4), 43.0 (C-3), 30.5 (C-6), 26.7 (C-5), 20.3 (C-8), 20.1 (C-9), 0.8 (C-10);
FTIR (ATR): $\tilde{\nu}$ /cm^{-1} = 1742 (C=O).

(±)-10-Iodocamphorquinone (9)

A suspension of **8** (13.9 g, 50 mmol) and freshly resublimed SeO_2 (6.9 g, 62 mmol) in bromobenzene (70 mL) was heated under reflux for 22 h. The cooled reaction mixture was concentrated at reduced pressure and filtered through a short silica column to remove arose selenium, the column was washed with ethyl acetate, and the solution was concentrated at reduced pressure. The residue was purified by column chromatography (silica gel, ethyl acetate/petroleum ether 1:5) to afford **9** as a yellow solid in 84% yield (12.25 g), mp 109–113 °C.

^{1}H NMR ($CDCl_3$): δ = 3.39 (d, J = 11.3 Hz, 1H, 10-H_a), 3.21 (d, J = 10.7 Hz, 1H, 10-H_b), 2.71 (d, J = 4.4 Hz, 1H, 4-H), 2.27–2.15 (m, 2H, CH_2), 1.92–1.82 (m, 1H, CH_2), 1.71–1.62 (m, 1H, CH_2), 1.18 (s, 3H, CH_3), 0.99 (s, 3H, CH_3);

^{13}C NMR ($CDCl_3$): δ = 200.8 (C═O), 200.5 (C═O), 60.0 (C-1), 58.7 (C-4), 43.7 (C-7), 30.7 (CH_2), 21.7 (CH_2), 21.3 (CH_3), 18.3 (CH_3), −3.0 (C-10);

FTIR (ATR): $\tilde{\nu}$/cm^{-1} = 1770 (C═O), 1751 (C═O);

MS: m/z (%) = 292 (2, M^+), 167 (1), 137 (71), 127 (5), 109 (100), 91 (10), 81 (23), 67 (70), 55 (23), 41 (26);

UV–vis ($CHCl_3$): λ_{max}/nm (ε/mol^{-1} dm^3 cm) = 467 (39).

(±)-10-Acetoxycamphorquinone (6) from 9

A suspension of **9** (12.0 g, 41.1 mmol), anhydrous acetic acid (22 mL) and freshly molten potassium acetate (30 g, 306 mmol) was heated at 170 °C for 8 h. The cooled reaction mixture was dissolved in water (100 mL), neutralized with an aqueous solution of Na_2CO_3 and extracted with diethyl ether (4 × 50 mL). Combined ethereal layers were washed with water (50 mL), dried over Na_2SO_4 and concentrated. The residue was purified by column chromatography (silica gel, ethyl acetate/petroleum ether 1:3) followed by crystallization (toluene/petroleum ether 1:3) to afford **6** as a yellow solid in 37% yield (3.4 g), mp 76–78 °C.

(±)-10-Methacryloyloxycamphorquinone (MCQ)

A solution of **7** (6.76 g, 37.1 mmol) and 2,6-di-*tert*-butyl-4-methylphenol (100 mg) in anhydrous dioxane (170 mL) was cooled to 0 °C under argon. A solution of triethylamine (5.1 mL, 37 mmol) and methacryloyl chloride (4 mL, 41 mmol) in anhydrous dioxane (50 mL) was added through a septum. The reaction mixture was

heated under reflux for 2 h, cooled to ambient temperature, filtered to remove solid triethylamine hydrochloride and concentrated at ambient temperature. The residue was dissolved in diethyl ether (200 mL) and water (6 mL). The water layer was removed, and the ethereal layer was dried over $MgSO_4$ and concentrated. The crude product was purified by column chromatography (silica gel, ethyl acetate/petroleum ether 1:5). Side-product **10** was removed by crystallization from diethyl ether, mp 132–134 °C. The yellow solid **MCQ** was obtained by crystallization from diethyl ether at −20°C in 42% yield (3.9 g), mp 46–49 °C. Solid **MCQ** was stored at +4 °C.

^1H NMR ($CDCl_3$): δ = 6.08 (s, 1H, vinyl-H_a), 5.59 (s, 1H, vinyl-H_b), 4.48 (d, J = 12.3 Hz, 1H, 10-H_a), 4.42 (d, J = 12.3 Hz, 1H, 10-H_b), 2.64 (d, J = 5.0 Hz, 1H, 4-H), 2.28–2.16 (m, 2H, 5-H + 6-H), 1.94 (s, 3H, methacrylic CH_3), 1.74–1.67 (m, 2H, 5-H + 6-H), 1.18 (s, 3H, 8-H), 1.09 (s, 3H, 9-H);

^{13}C NMR ($CDCl_3$): δ = 201.5 (C═O), 201.1 (C═O), 167.0 (O─<u>C</u>═O), 135.8 (<u>C</u>═CH_2), 126.2 (C═<u>C</u>H_2), 61.1 (C-1), 59.5 (C-10), 58.6 (C-4), 42.8 (C-7), 25.9 (C-6), 22.1 (C-9), 21.8 (C-5), 18.3 (C-8), 18.3 (methacrylic CH_3);

FTIR (ATR): $\tilde{\nu}$/cm^{-1} = 3100 (vinyl C─H), 1774 (C═O), 1755 (C═O), 1727 (methacrylic C═O), 1641 (C═C);

MS: m/z (%) = 250 (1, $M^{+\cdot}$), 222 (9), 207 (2), 153 (10), 136 (32), 121 (10), 108 (20), 93 (33), 79 (17), 69 (100), 55 (22), 41 (33);

UV–vis ($CHCl_3$): λ_{max}/nm (ε/mol^{-1} dm^3 cm) = 466 (39).

(7,7-Dimethyl-2,3-dioxobicyclo[2.2.1]hept-1-yl)methyl 6-chloro-2,5-dimethyl-3,4-dihydro-2*H*-pyran-2-carboxylate (10)

^{1}H NMR ($CDCl_3$): δ = 4.49 (d, J = 12.2 Hz, 1H, $C\underline{H}_2O$), 4.36 (d, J = 12.2 Hz, 1H, $C\underline{H}_2O$), 2.65 (d, J = 4.6 Hz, 1H, $C\underline{H}$–C=O), 2.31 (ddd, J = 13.4, 6.4, 2.1 Hz, 1H, $C\underline{H}_2$–C–O), 2.25–2.07 (m, 3H, $C\underline{H}_2$–$C\underline{H}_2$–CH + $C\underline{H}_2$–C=C), 2.01 (d, J = 5.5 Hz, 1H, $C\underline{H}_2$–C=C), 1.79 (ddd, J = 13.4, 11.0, 6.4 Hz, 1H, $C\underline{H}_2$–C–O), 1.74–1.63 (m, 5H, C=C–$C\underline{H}_3$ + $C\underline{H}_2$–$C\underline{H}_2$–CH), 1.53 (s, 3H, $C\underline{H}_3$–C–O), 1.20 (s, 3H, CH_3–C–$C\underline{H}_3$), 1.09 (s, 3H, CH_3–C–$C\underline{H}_3$);

^{13}C NMR ($CDCl_3$): δ = 201.4 ($\underline{C}$=O), 200.9 ($\underline{C}$=O), 172.5 (O–$\underline{C}$=O), 136.1 (C=$\underline{C}$–Cl), 103.7 (C=$\underline{C}$–CH_3), 81.1 (CH_3–$\underline{C}$–O), 60.8 ($\underline{C}$–CH_2O), 60.4 ($\underline{C}H_2$–O), 58.4 ($\underline{C}$H–C=O), 42.8 (CH_3–$\underline{C}$–CH_3), 30.6 ($\underline{C}H_2$–C–O), 25.6 ($\underline{C}H_2$–CH_2–CH), 25.4 ($\underline{C}H_2$–C=C), 25.0 ($\underline{C}H_3$–C–O), 22.0 (CH_3–C–$\underline{C}H_3$), 21.8(CH_2–$\underline{C}H_2$–CH), 18.4 (CH_3–C–$\underline{C}H_3$), 18.2 (C=C–$\underline{C}H_3$);

FTIR (ATR): $\tilde{\nu}$ /cm^{-1} = 1776 (C=O), 1759 (C=O), 1749 (esteric C=O), 1677 (C=C)

UV–vis ($CHCl_3$): λ_{max}/nm (ε/mol^{-1} dm^3 cm) = 467 (35).

Polymerization

A mixture of **MCQ** (0.199 g, 0.80 mmol), styrene (20 mL, 174 mmol), and AIBN (20 mg) was sealed under argon in an ampoule and polymerized at 59 ºC for 4.5 h. The copolymer **MCQ/S** was precipitated three times from chloroform solution into methanol. The conversion was 10% (1.84 g). The content of **MCQ** units in **MCQ/S** copolymer was determined to be 0.72 mol % from FTIR spectroscopy and 0.62 mol% from UV–vis spectroscopy.

GPC: $M_n = 1.7 \times 10^5$ g mol^{-1}, $M_w = 3.4 \times 10^5$ g mol^{-1};

FTIR (film): $\tilde{\nu}$ /cm^{-1} = 1776 (C=O), 1759 (C=O), 1732 (methacrylic C=O).

Irradiation

Films of **MCQ/S** copolymer (40 mg) with good optical quality about 40 µm thick were prepared by casting from 1 mL benzene solutions onto a glass plate (10 cm^2 area). The plate was covered by a Petri dish to slow down the evaporation of the solvent. The self-supporting polymer films were separated from the glass by dipping into distilled water. Films were dried to a constant weight at room temperature under vacuum and were irradiated at λ > 380 nm at ambient temperature in air. A homemade carousel apparatus was employed. It consisted of a 125 W medium-pressure mercury arc placed in a circulating-water-jacketed quartz tube, which was surrounded by a 1 cm thick layer of a liquid filter (500 g NaBr and 3 g $Pb(NO_3)_2$ per 1000 mL aqueous solution) transmitting at λ > 330 nm. The distance of each sample (placed in rotating eight-rectangular holders) from the arc was about 8 cm. Finally, a plastic filter UV CL SR HPR (LLumar, USA) transmitting at λ > 380 nm was placed in front of the sample.
