# Supplementary material for: Synthesis and photooxidation of styrene copolymer bearing camphorquinone pendant groups
Source: Beilstein J Org Chem. 2012 Mar 6;8:337–43. doi: 10.3762/bjoc.8.37 (PMC3326610; doi:10.3762/bjoc.8.37)
Supplement: File 2 — NMR spectra of compounds 5–10 and MCQ. [file Beilstein_J_Org_Chem-08-337-s002.pdf]

Supporting Information File 2

for

Synthesis and photooxidation of styrene copolymer bearing camphorquinone pendant groups

Branislav Husár*[1], Norbert Moszner[2] and Ivan Lukáč[1]

Address: [1]Polymer Institute, Slovak Academy of Sciences, Dúbravská cesta 9, SK-845 41 Bratislava 45, Slovakia and [2]Ivoclar Vivadent AG, Bendererstrasse 2, FL-9494 Schaan, Liechtenstein

Email: Branislav Husár* - branislav.husar@savba.sk; Norbert Moszner - norbert.moszner@ivoclarvivadent.com; Ivan Lukáč - ivan.lukac@savba.sk

* Corresponding author

NMR spectra of compounds 5–10 and MCQ

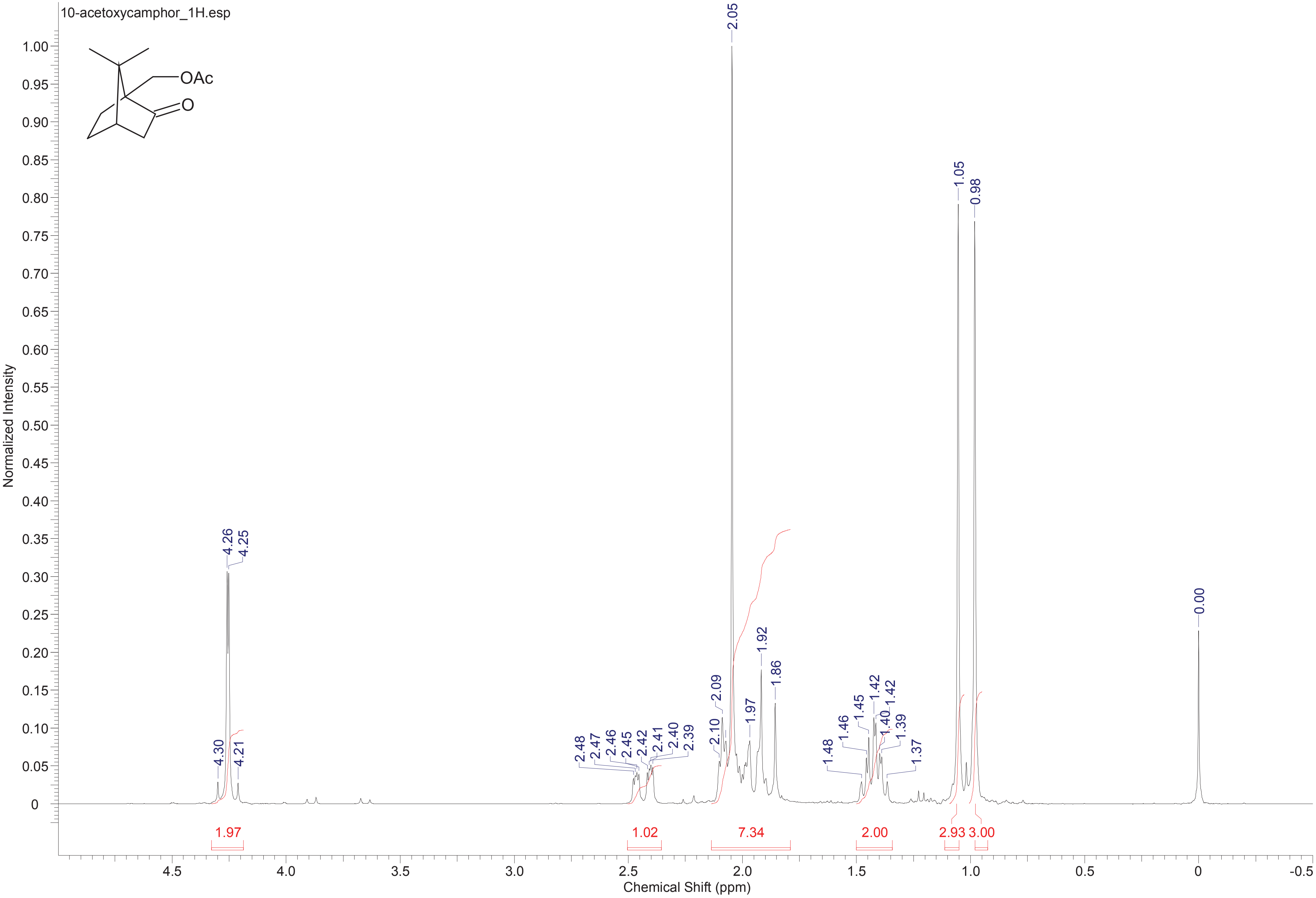
10-acetoxycamphor_1H.esp
OAc
O
Normalized Intensity
Chemical Shift (ppm)
4.30
4.26
4.25
4.21
2.48
2.47
2.46
2.45
2.42
2.41
2.40
2.39
2.10
2.09
2.05
1.97
1.92
1.86
1.48
1.46
1.45
1.42
1.42
1.40
1.39
1.37
1.05
0.98
0.00
1.97
1.02
7.34
2.00
2.93
3.00

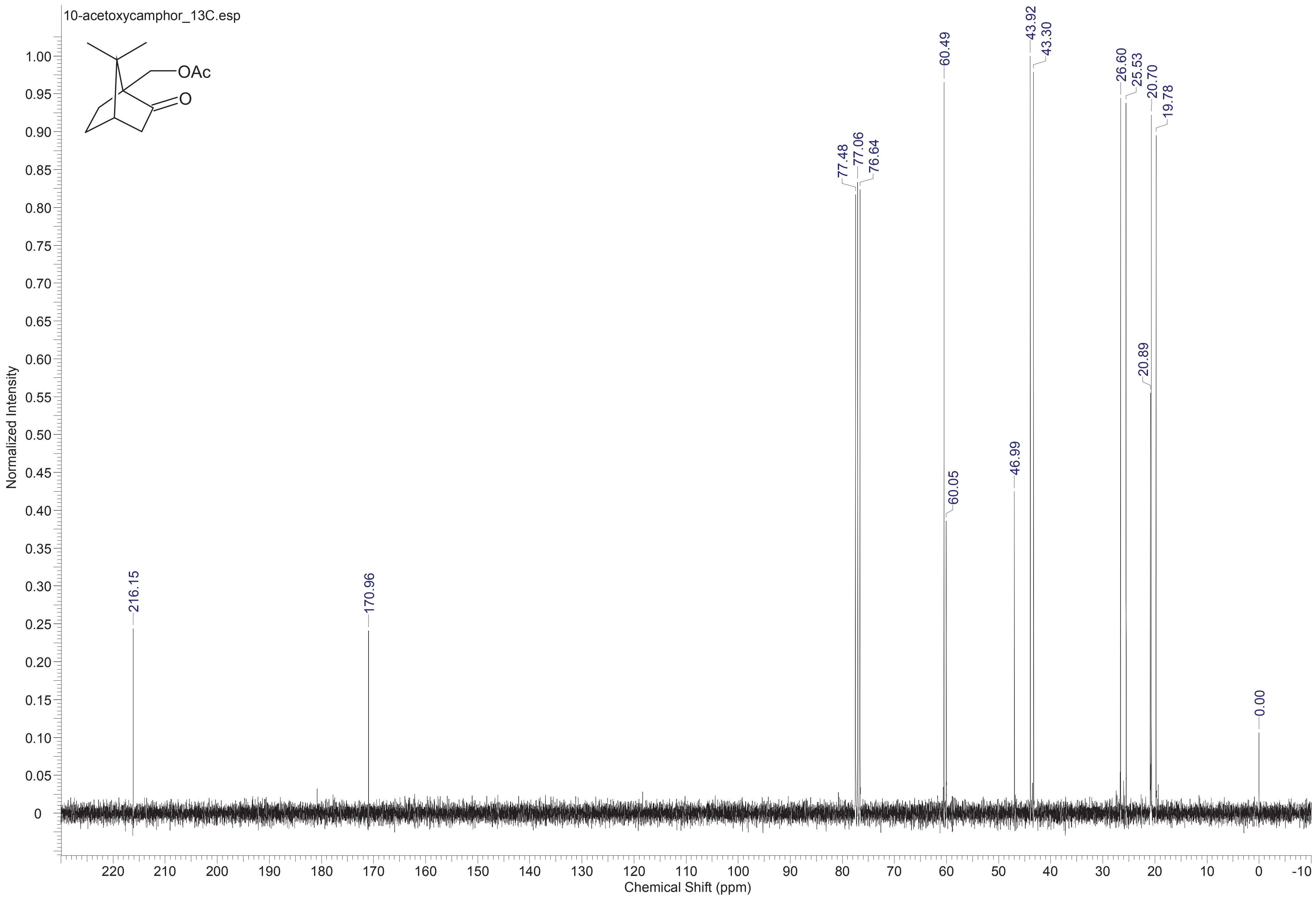

10-acetoxycamphor_13C.esp
OAc
O
216.15
170.96
77.48
77.06
76.64
60.49
60.05
46.99
43.92
43.30
26.60
25.53
20.89
20.70
19.78
0.00
Normalized Intensity
Chemical Shift (ppm)

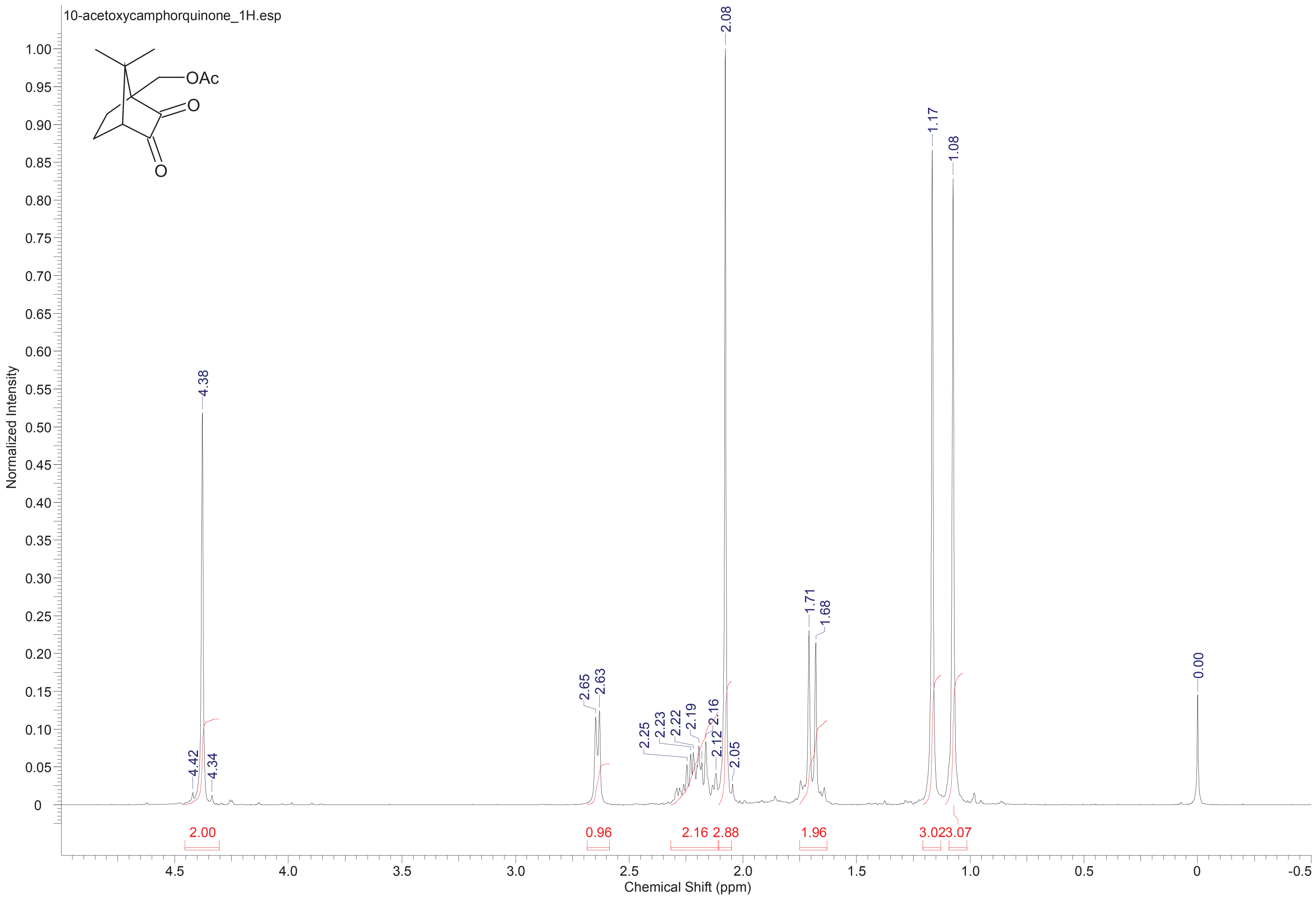
10-acetoxycamphorquinone_1H.esp
OAc
O
O
Normalized Intensity
Chemical Shift (ppm)
4.42
4.38
4.34
2.65
2.63
2.25
2.23
2.22
2.19
2.16
2.12
2.08
2.05
1.71
1.68
1.17
1.08
0.00
2.00
0.96
2.16
2.88
1.96
3.02
3.07

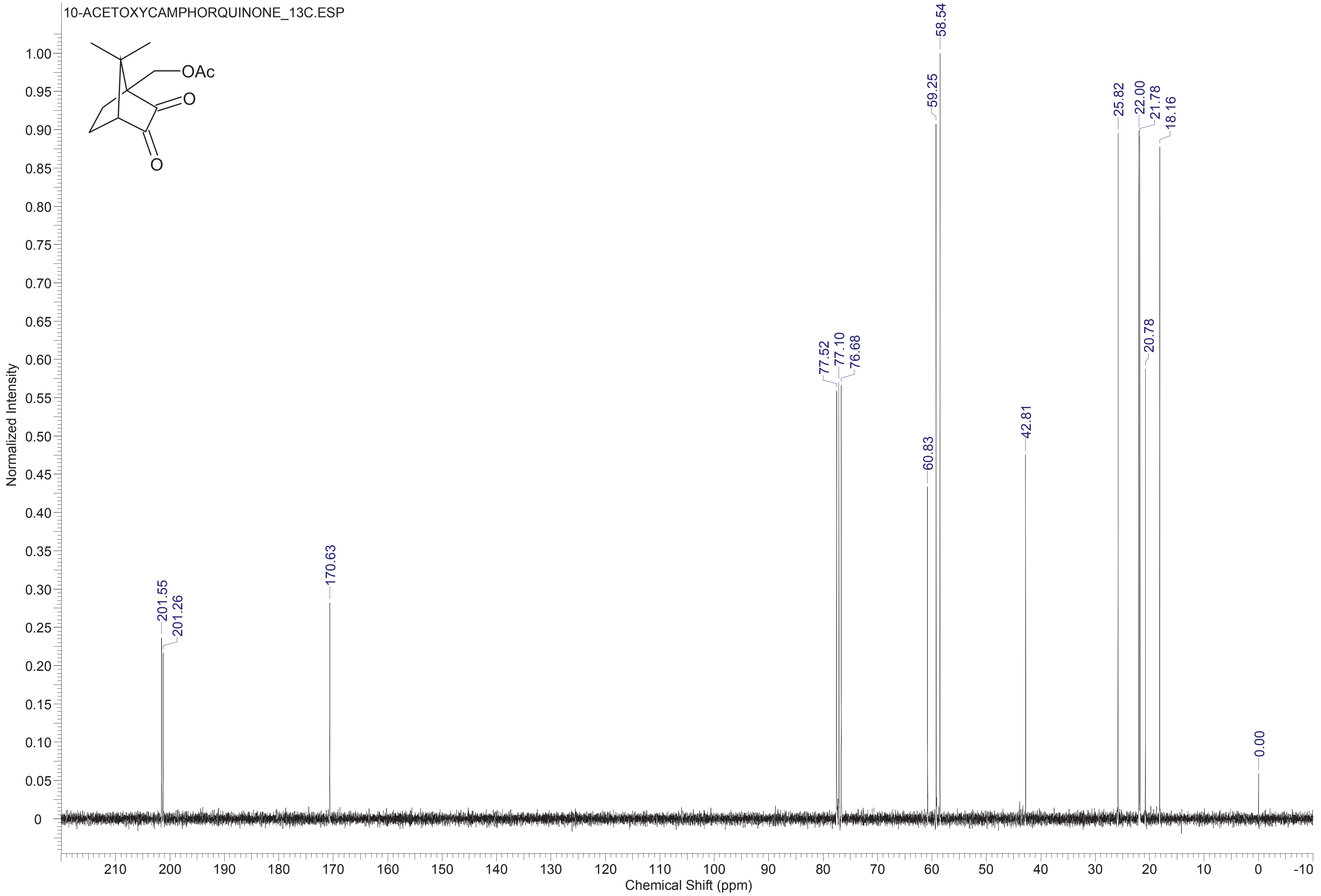
10-ACETOXYCAMPHORQUINONE_13C.ESP
OAc
O
O
201.55
201.26
170.63
77.52
77.10
76.68
60.83
59.25
58.54
42.81
25.82
22.00
21.78
20.78
18.16
0.00
Normalized Intensity
Chemical Shift (ppm)

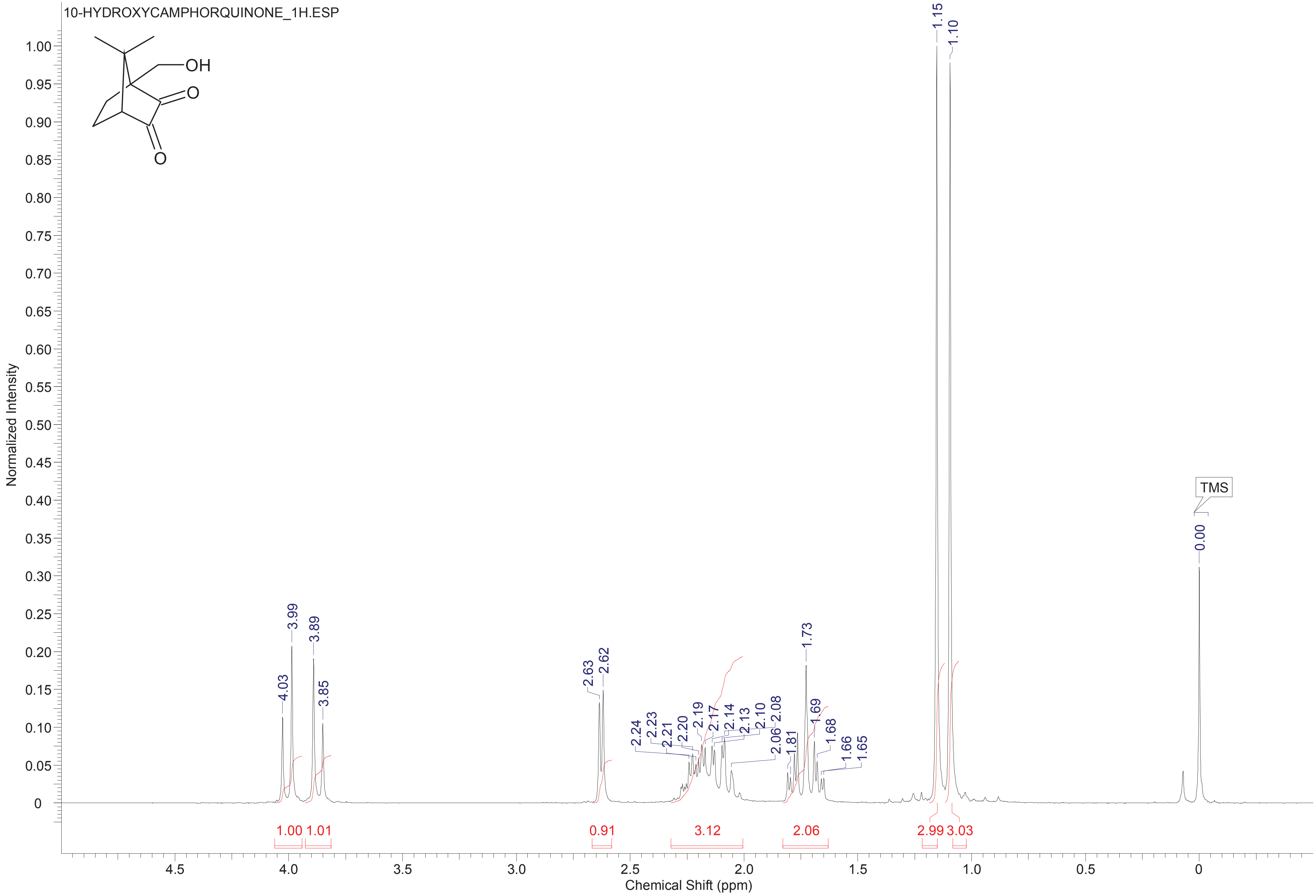

10-HYDROXYCAMPHORQUINONE_1H.ESP
OH
O
O
Normalized Intensity
1.00
0.95
0.90
0.85
0.80
0.75
0.70
0.65
0.60
0.55
0.50
0.45
0.40
0.35
0.30
0.25
0.20
0.15
0.10
0.05
0
4.03
3.99
3.89
3.85
2.63
2.62
2.24
2.23
2.21
2.20
2.19
2.17
2.14
2.13
2.10
2.08
2.06
1.81
1.73
1.69
1.68
1.66
1.65
1.15
1.10
TMS
0.00
1.00
1.01
0.91
3.12
2.06
2.99
3.03
4.5
4.0
3.5
3.0
2.5
2.0
1.5
1.0
0.5
0
Chemical Shift (ppm)

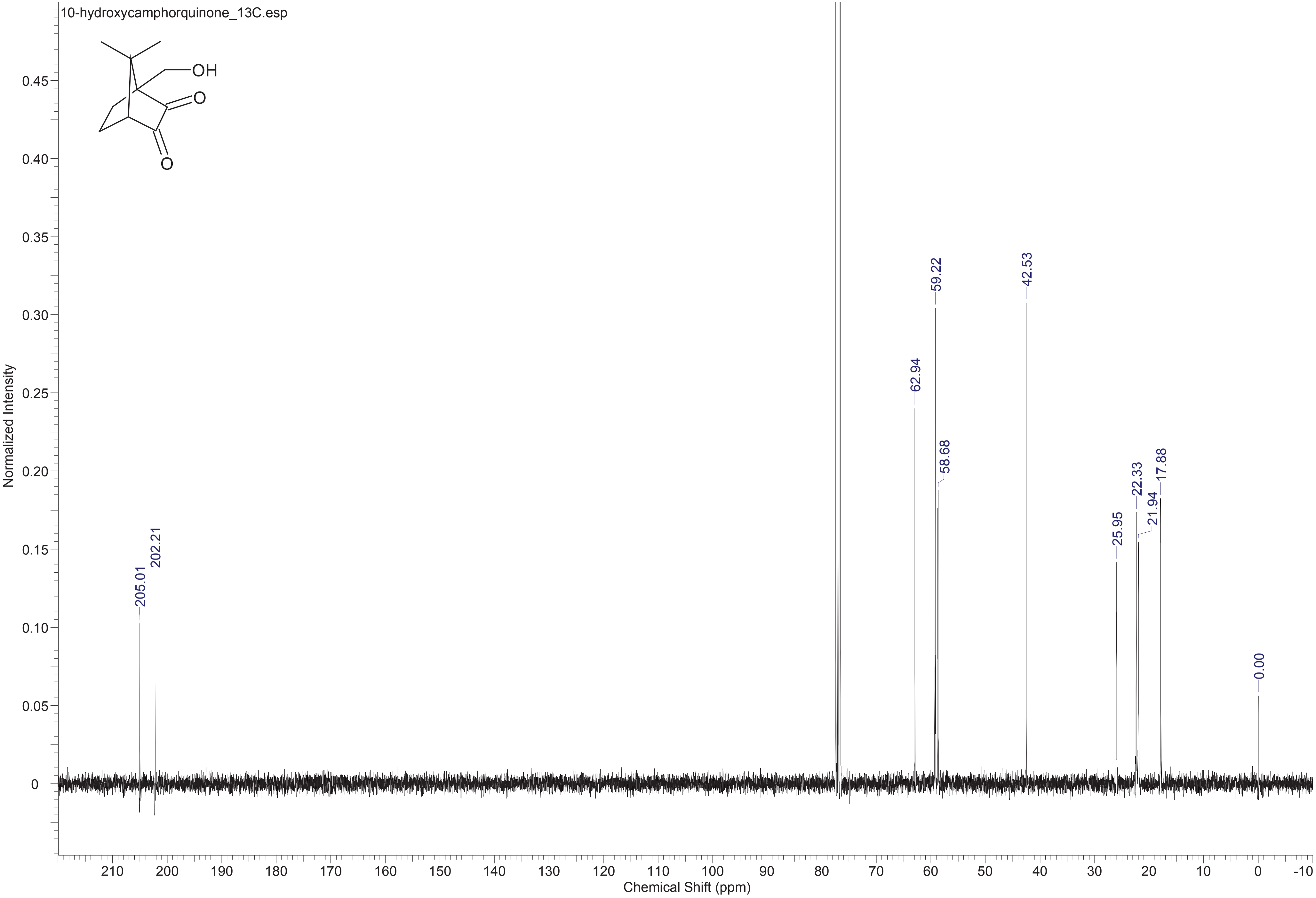

10-hydroxycamphorquinone_13C.esp
OH
O
O
205.01
202.21
62.94
59.22
58.68
42.53
25.95
22.33
21.94
17.88
0.00
Normalized Intensity
0.45
0.40
0.35
0.30
0.25
0.20
0.15
0.10
0.05
0
210 200 190 180 170 160 150 140 130 120 110 100 90 80 70 60 50 40 30 20 10 0 -10
Chemical Shift (ppm)

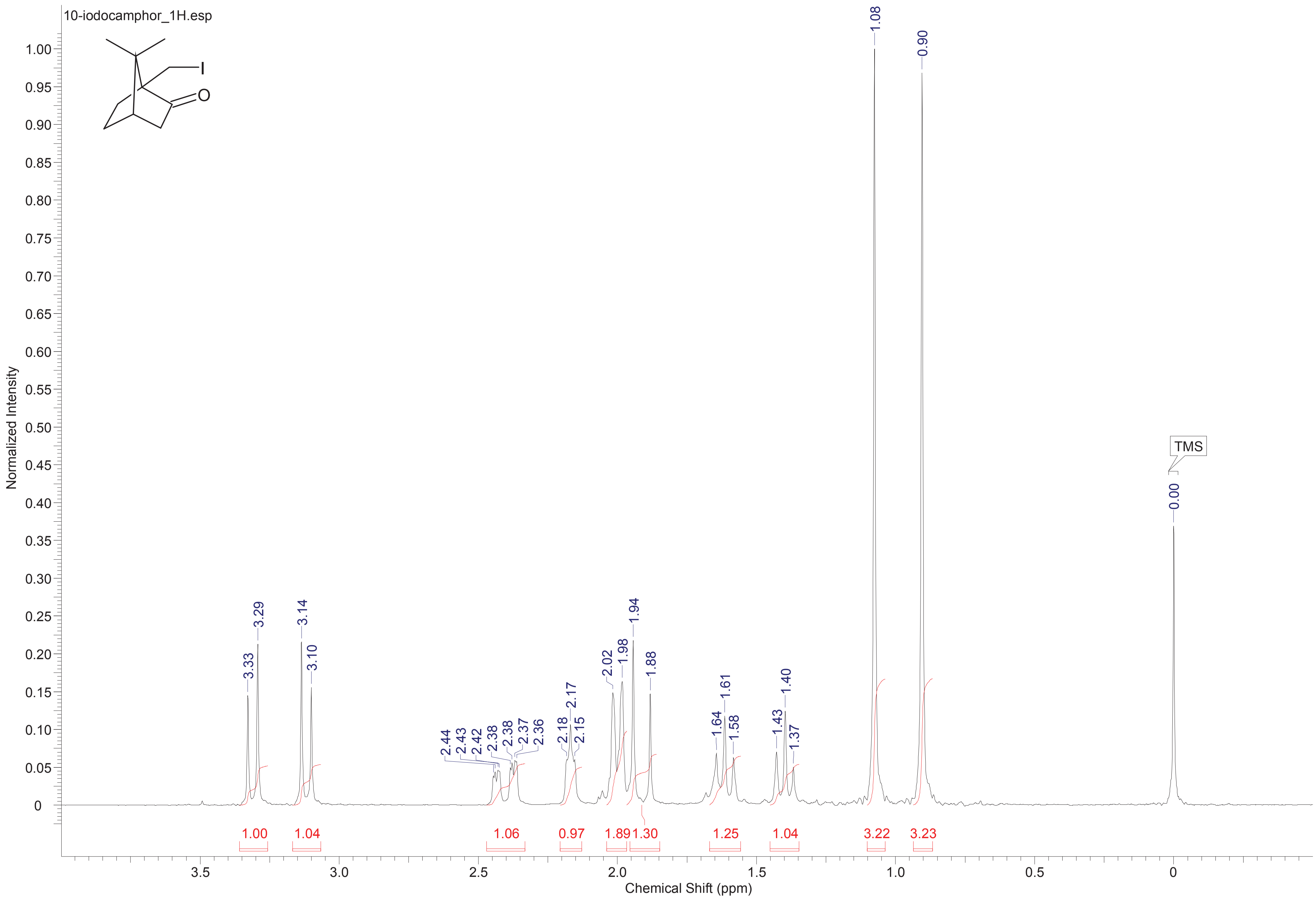
10-iodocamphor_1H.esp
I
O
TMS
0.00
0.90
1.08
1.37
1.40
1.43
1.58
1.61
1.64
1.88
1.94
1.98
2.02
2.15
2.17
2.18
2.36
2.37
2.38
2.38
2.42
2.43
2.44
3.10
3.14
3.29
3.33
1.00
1.04
1.06
0.97
1.89
1.30
1.25
1.04
3.22
3.23
Normalized Intensity
Chemical Shift (ppm)

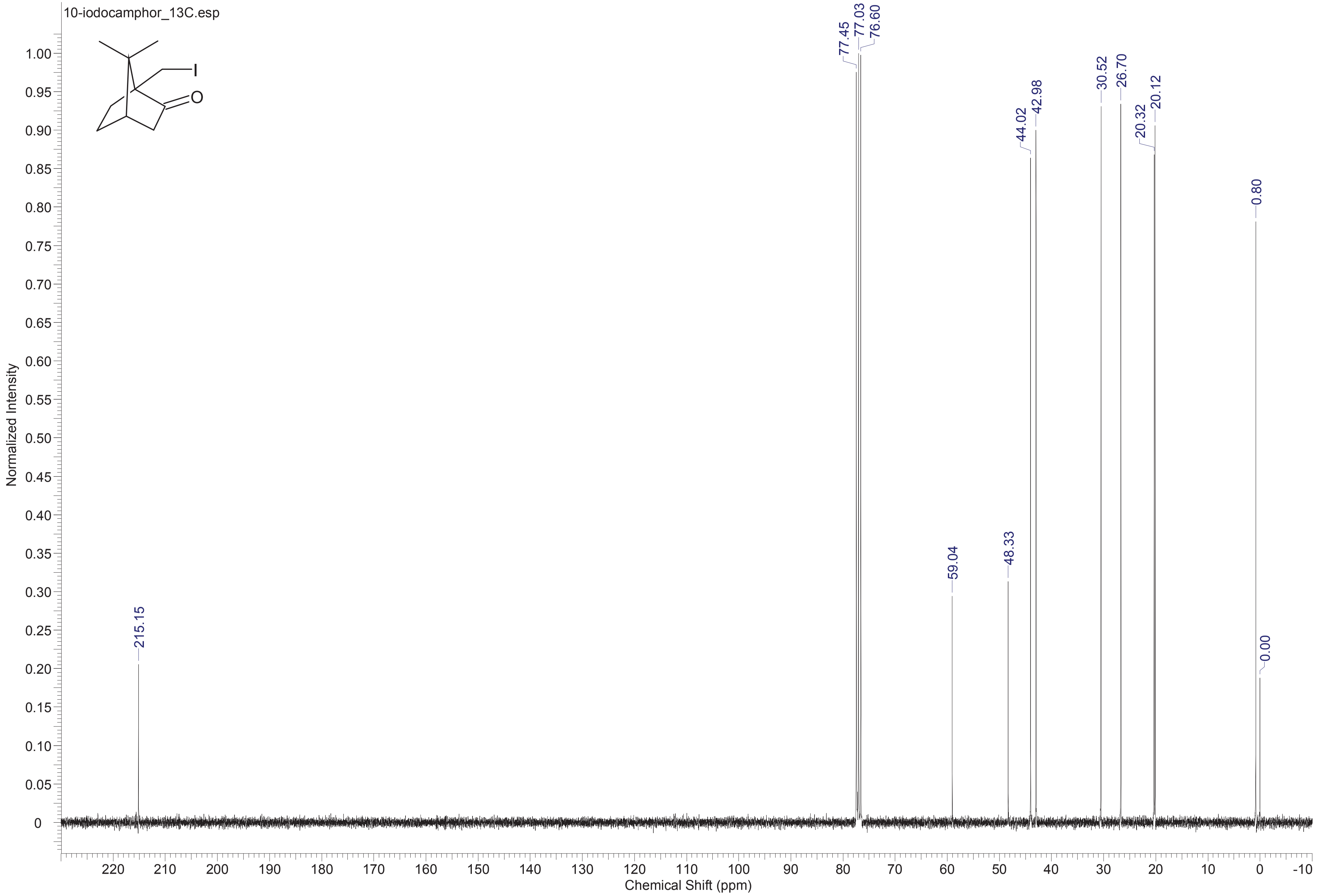

10-iodocamphor_13C.esp
I
O
77.45
77.03
76.60
30.52
26.70
44.02
42.98
20.32
20.12
0.80
59.04
48.33
215.15
0.00
Normalized Intensity
1.00
0.95
0.90
0.85
0.80
0.75
0.70
0.65
0.60
0.55
0.50
0.45
0.40
0.35
0.30
0.25
0.20
0.15
0.10
0.05
0
220
210
200
190
180
170
160
150
140
130
120
110
100
90
80
70
60
50
40
30
20
10
0
-10
Chemical Shift (ppm)

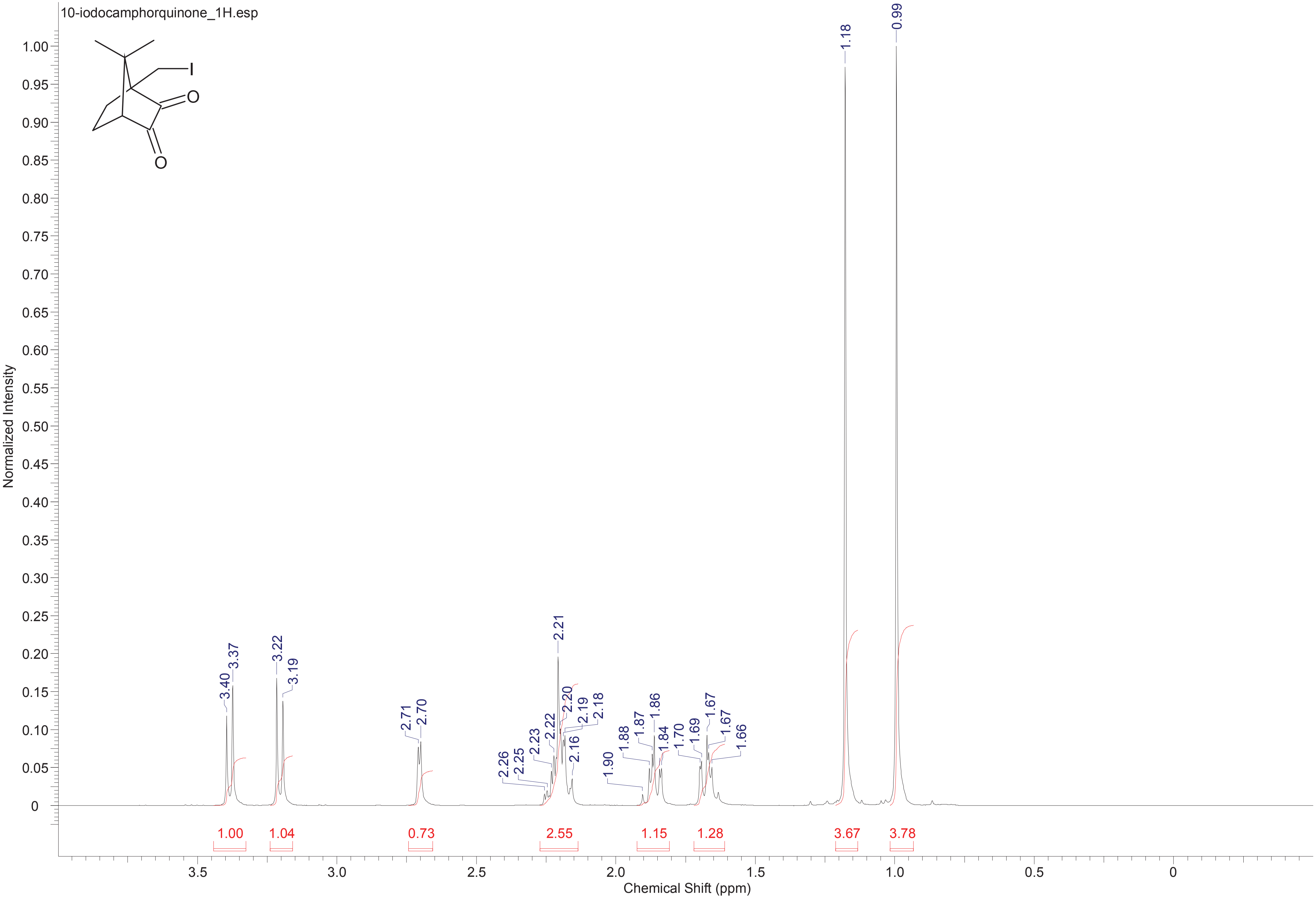
10-iodocamphorquinone_1H.esp
O
O
I
Normalized Intensity
1.00
0.95
0.90
0.85
0.80
0.75
0.70
0.65
0.60
0.55
0.50
0.45
0.40
0.35
0.30
0.25
0.20
0.15
0.10
0.05
0
3.40
3.37
3.22
3.19
2.71
2.70
2.26
2.25
2.23
2.22
2.21
2.20
2.19
2.18
2.16
1.90
1.88
1.87
1.86
1.84
1.70
1.69
1.67
1.67
1.66
1.18
0.99
1.00
1.04
0.73
2.55
1.15
1.28
3.67
3.78
3.5
3.0
2.5
2.0
1.5
1.0
0.5
0
Chemical Shift (ppm)

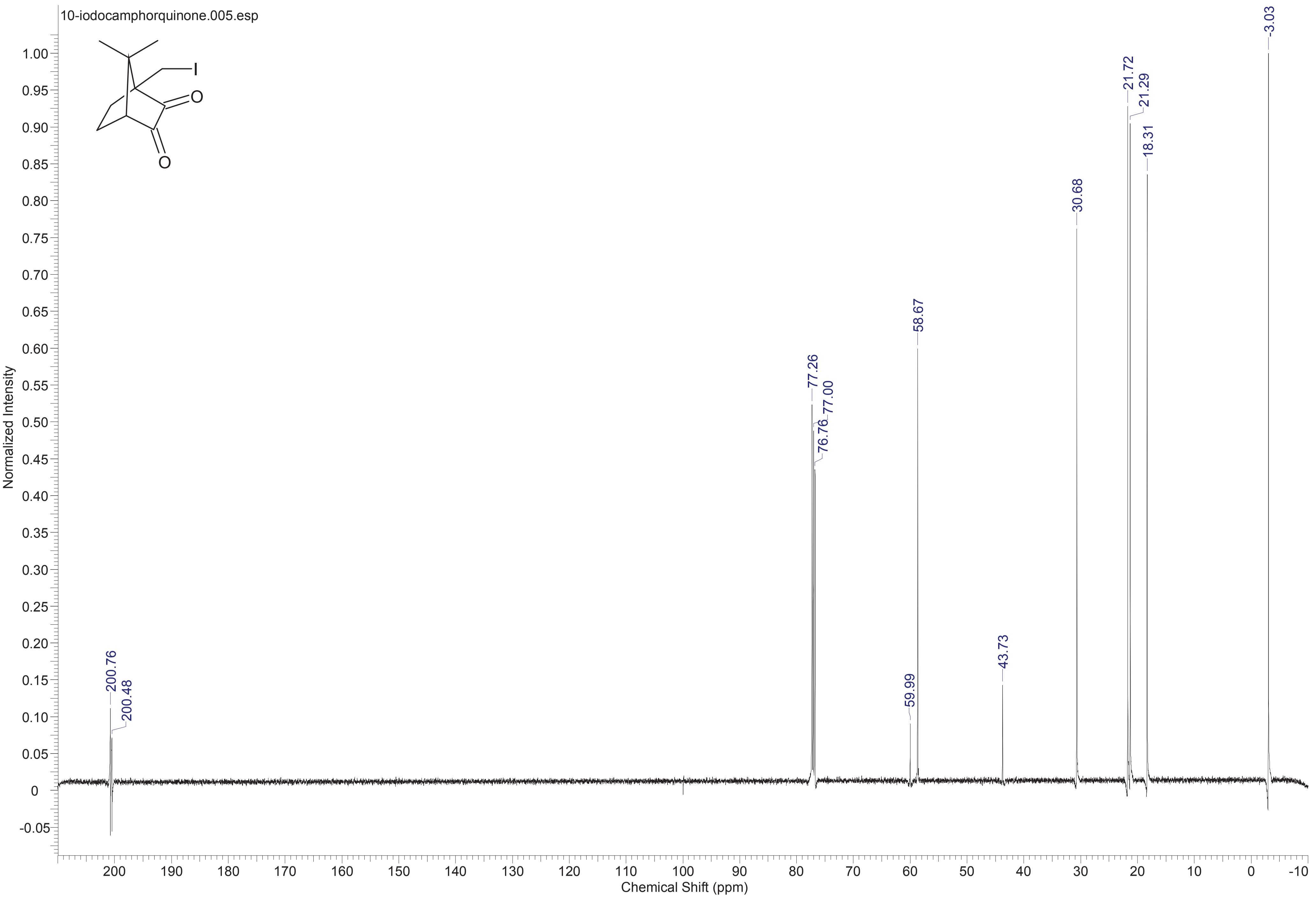
10-iodocamphorquinone.005.esp
I
O
O
Normalized Intensity
Chemical Shift (ppm)
200.76
200.48
77.26
77.00
76.76
59.99
58.67
43.73
30.68
21.72
21.29
18.31
-3.03

10-iodocamphorquinone_COSY.esp

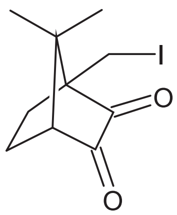

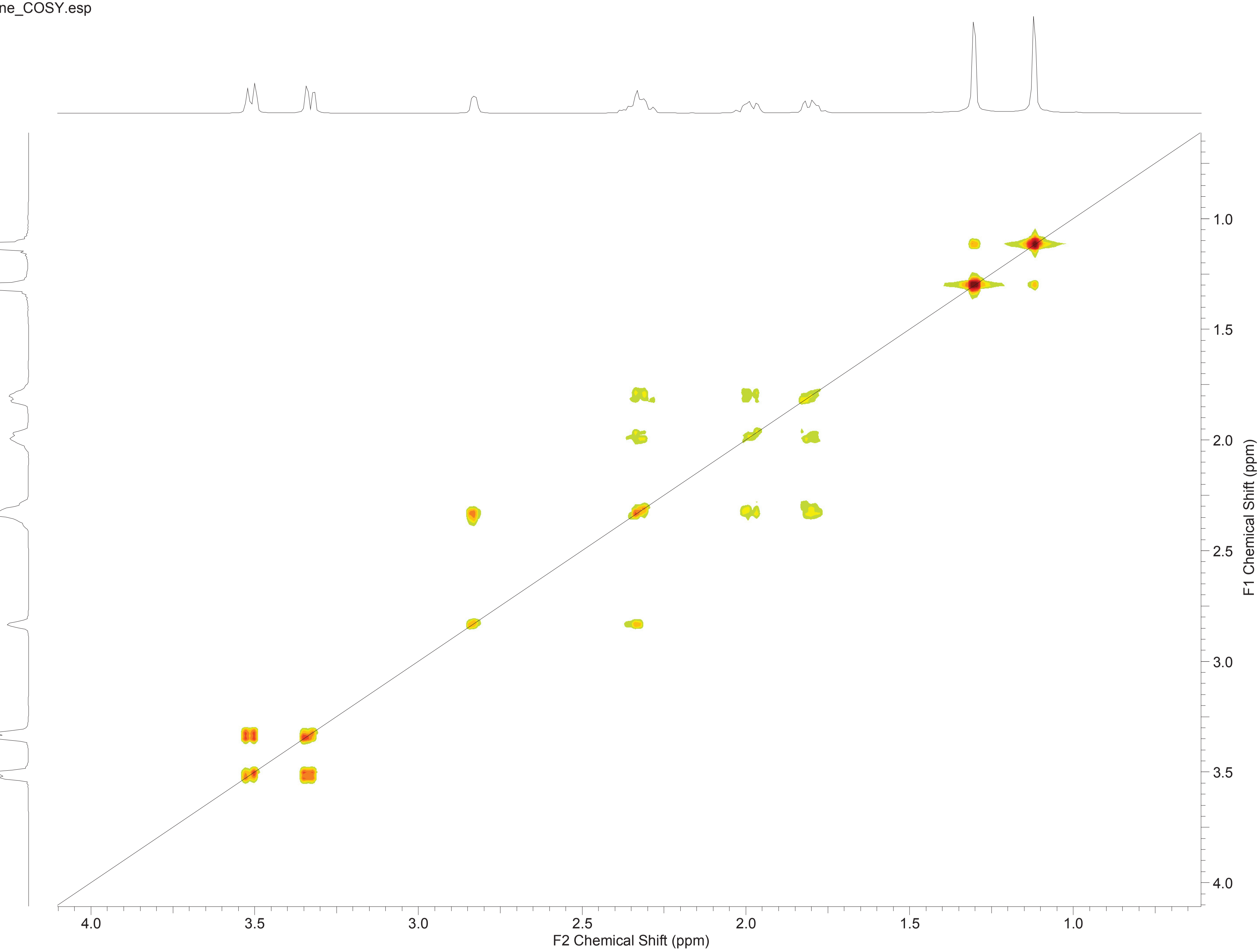

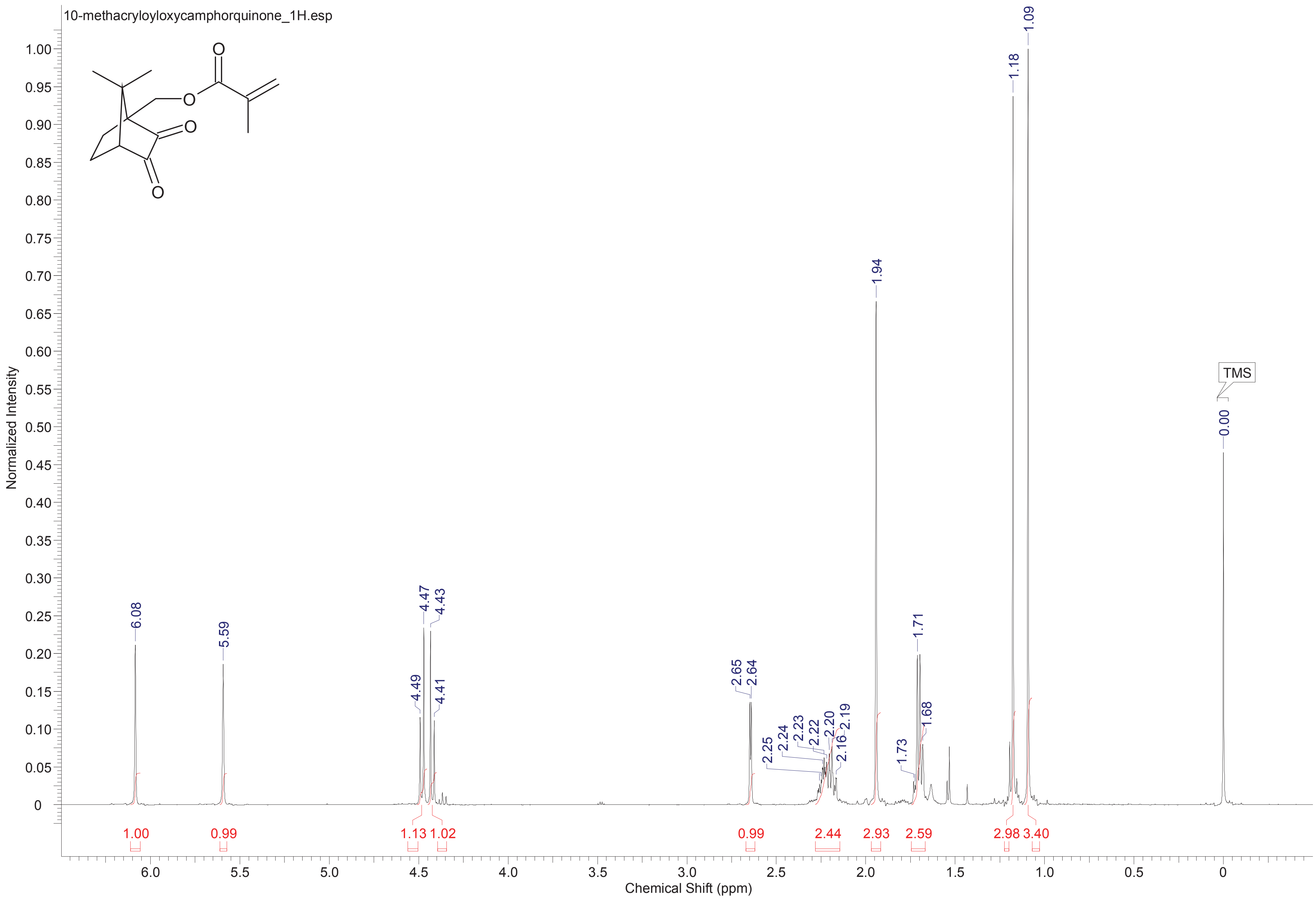
10-methacryloyloxycamphorquinone_1H.esp
Normalized Intensity
Chemical Shift (ppm)
TMS
6.08
5.59
4.49
4.47
4.43
4.41
2.65
2.64
2.25
2.24
2.23
2.22
2.20
2.19
2.16
1.94
1.73
1.71
1.68
1.18
1.09
0.00
1.00
0.99
1.13
1.02
0.99
2.44
2.93
2.59
2.98
3.40

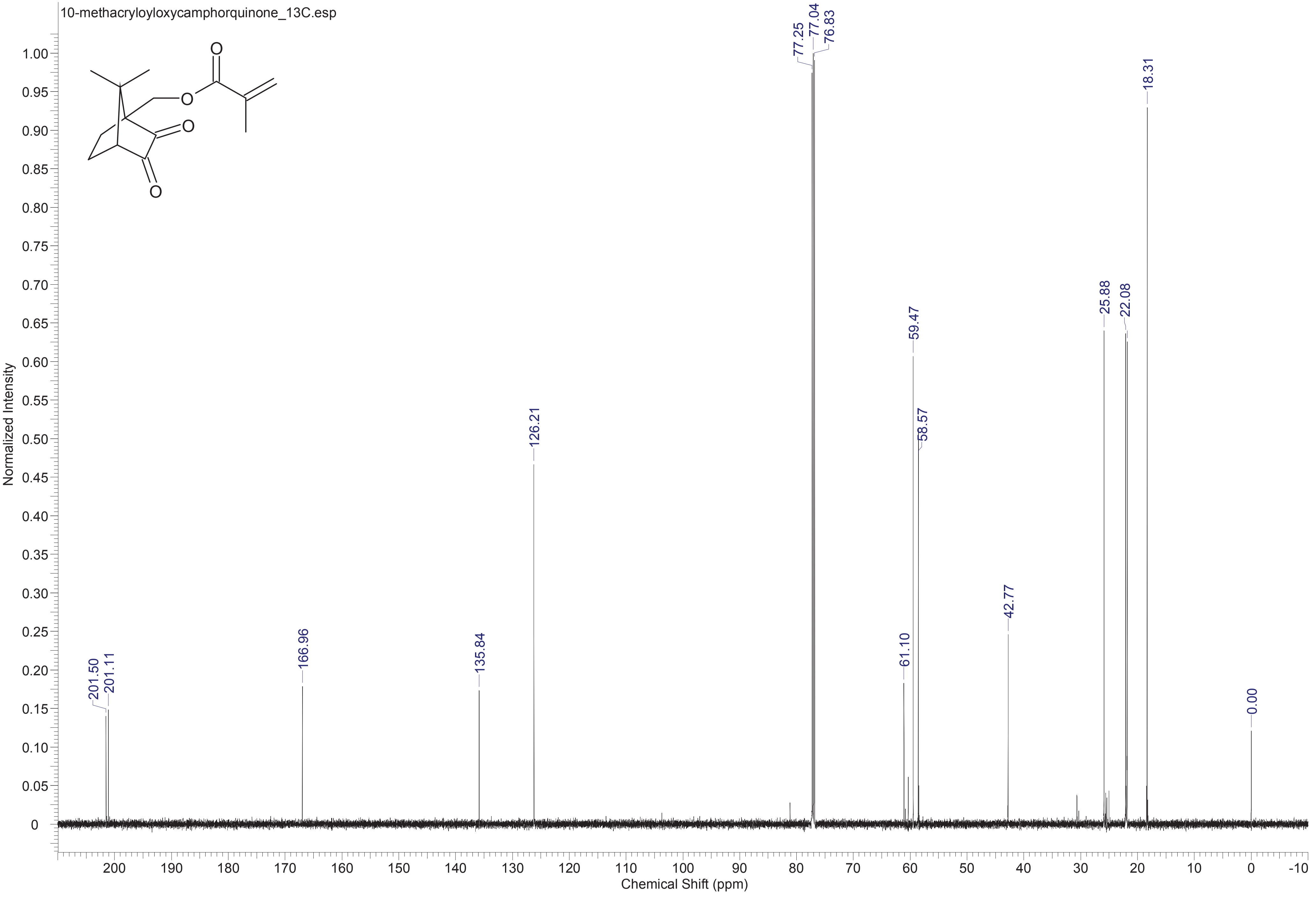
10-methacryloyloxycamphorquinone_13C.esp
201.50
201.11
166.96
135.84
126.21
77.25
77.04
76.83
61.10
59.47
58.57
42.77
25.88
22.08
18.31
0.00
Normalized Intensity
Chemical Shift (ppm)

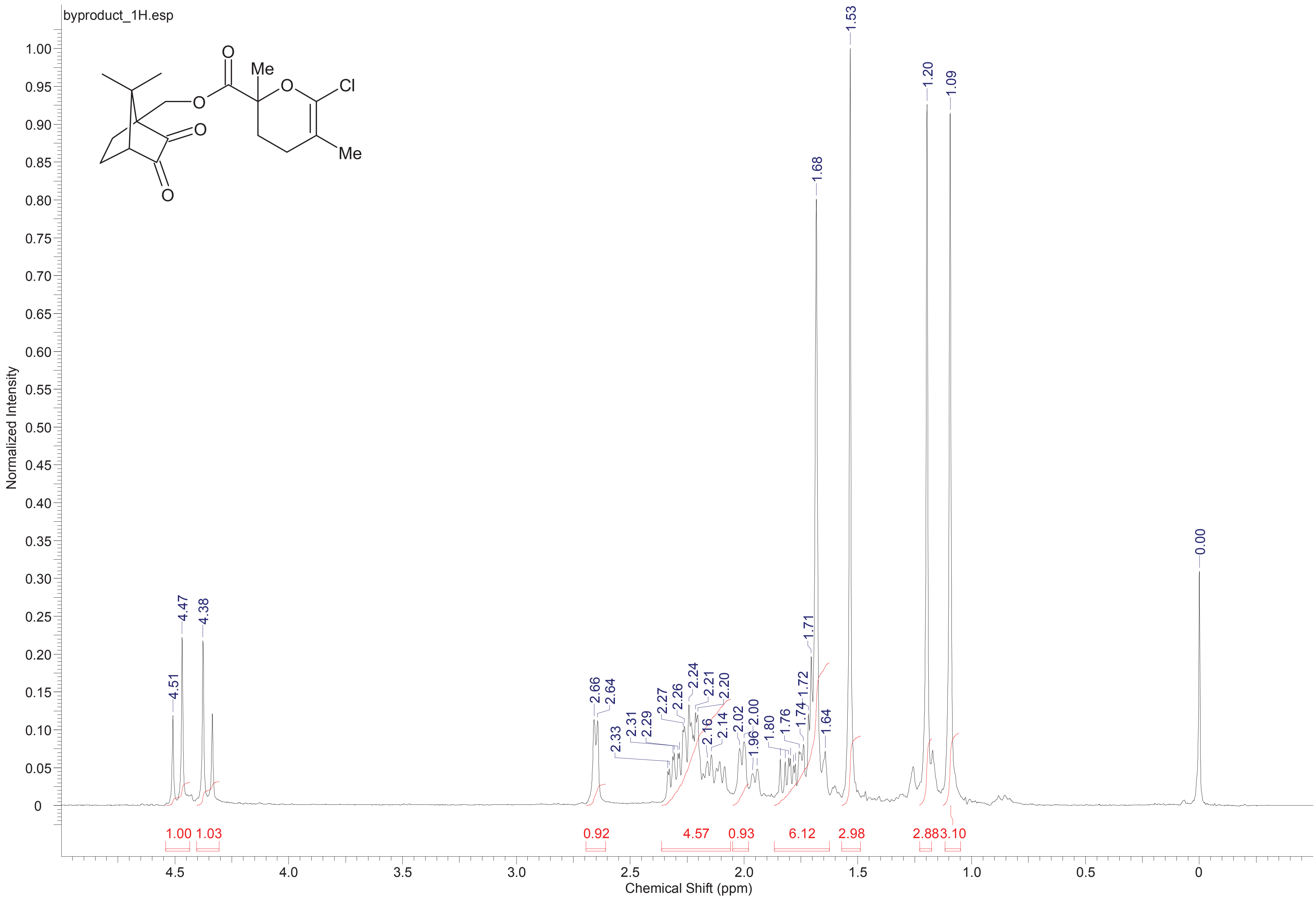

byproduct_1H.esp
Me
O
O
O
O
O
Cl
Me
Normalized Intensity
Chemical Shift (ppm)
4.51
4.47
4.38
2.66
2.64
2.33
2.31
2.29
2.27
2.26
2.24
2.21
2.20
2.16
2.14
2.02
2.00
1.96
1.80
1.76
1.74
1.72
1.71
1.68
1.64
1.53
1.20
1.09
0.00
1.00
1.03
0.92
4.57
0.93
6.12
2.98
2.88
3.10

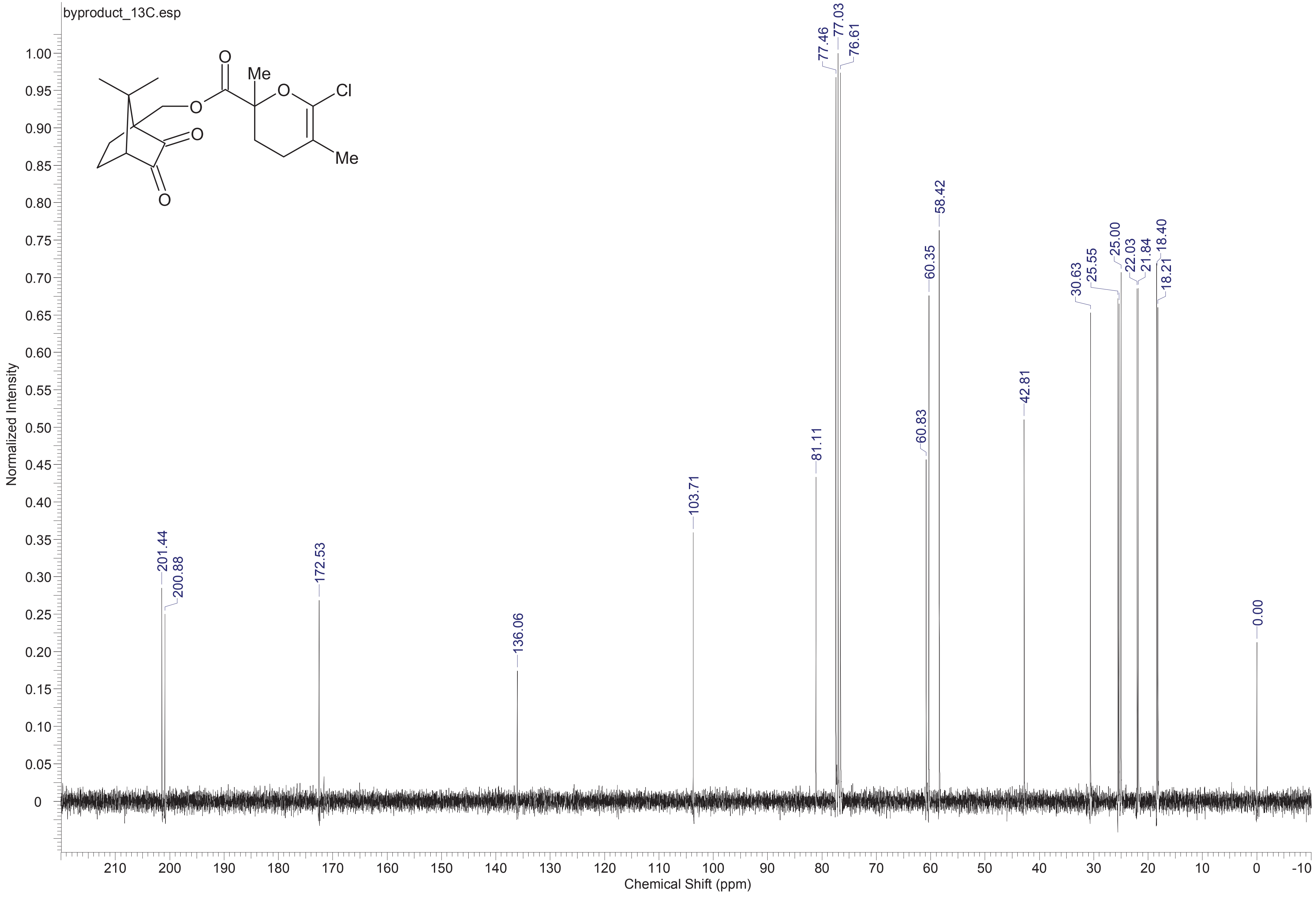

byproduct_13C.esp
Me
O
Cl
Me
O
O
O
O
201.44
200.88
172.53
136.06
103.71
81.11
77.46
77.03
76.61
60.83
60.35
58.42
42.81
30.63
25.55
25.00
22.03
21.84
18.40
18.21
0.00
Normalized Intensity
Chemical Shift (ppm)
